# H/ACA snRNP–dependent ribosome biogenesis regulates translation of polyglutamine proteins

**DOI:** 10.1126/sciadv.ade5492

**Published:** 2023-06-21

**Authors:** Shane M. Breznak, Yingshi Peng, Limin Deng, Noor M. Kotb, Zachary Flamholz, Ian T. Rapisarda, Elliot T. Martin, Kara A. LaBarge, Dan Fabris, Elizabeth R. Gavis, Prashanth Rangan

**Affiliations:** ^1^ Department of Biological Sciences, RNA Institute, University at Albany, 1400 Washington Avenue, LSRB 2033D, Albany, NY 12222, USA.; ^2^ Department of Molecular Biology, Princeton University, Princeton, NJ 08544, USA.; ^3^ Department of Chemistry, University of Connecticut, 55N Eagleville Rd, Storrs, CT 06269, USA.; ^4^ Department of Biomedical Sciences, University at Albany School of Public Health, Albany, NY 12144, USA.; ^5^ Lake Erie College of Osteopathic Medicine, College of Medicine, 1858 W Grandview Blvd, Erie, PA 16509, USA.; ^6^ Black Family Stem Cell Institute, Department of Cell, Developmental, and Regenerative Biology, Icahn School of Medicine at Mount Sinai, 1 Gustave L. Levy Place, New York, NY 10029, USA.

## Abstract

Stem cells in many systems, including *Drosophila* germline stem cells (GSCs), increase ribosome biogenesis and translation during terminal differentiation. Here, we show that the H/ACA small nuclear ribonucleoprotein (snRNP) complex that promotes pseudouridylation of ribosomal RNA (rRNA) and ribosome biogenesis is required for oocyte specification. Reducing ribosome levels during differentiation decreased the translation of a subset of messenger RNAs that are enriched for CAG trinucleotide repeats and encode polyglutamine-containing proteins, including differentiation factors such as RNA-binding Fox protein 1. Moreover, ribosomes were enriched at CAG repeats within transcripts during oogenesis. Increasing target of rapamycin (TOR) activity to elevate ribosome levels in H/ACA snRNP complex–depleted germlines suppressed the GSC differentiation defects, whereas germlines treated with the TOR inhibitor rapamycin had reduced levels of polyglutamine-containing proteins. Thus, ribosome biogenesis and ribosome levels can control stem cell differentiation via selective translation of CAG repeat–containing transcripts.

## Teaser

Translation of polyglutamine proteins requires H/ACA snRNP-dependent ribosome biogenesis.

## INTRODUCTION

Understanding how stem cells self-renew and differentiate is crucial to understanding the mechanisms of development and disease (*1*). Defects in ribosome biogenesis can impair stem cell differentiation and lead to diseases collectively called ribosomopathies (*2*–*5*). Protein synthesis often increases during stem cell differentiation (*6*–*8*), and inhibiting translation by modulating target of rapamycin (TOR) activity blocks terminal differentiation of various stem cells (*6*, *8*–*10*). Nevertheless, how ribosome levels and translation control differentiation remains incompletely understood. In the ribosomopathy Diamond-Blackfan anemia, mutations in ribosomal proteins limit the pool of available ribosomes, which alters the translation of a select subset of transcripts in hematopoietic stem and progenitor cells, leading to impaired erythroid lineage commitment (*11*).

RNAs are extensively modified by posttranscriptional modifications (PTMs), including pseudouridylation (*12*, *13*). The ribosomal RNA (rRNA) pseudouridine synthase subunit DKC1 is mutated in the ribosomopathy X-linked dyskeratosis congenita, an inherited bone marrow failure syndrome that is sometimes associated with impaired neurodevelopment (*14*). DKC1 is a member of the small nucleolar RNA–guided H/ACA small nuclear ribonucleoprotein (snRNP) complex, which deposits pseudouridine on rRNA at functionally important sites of the ribosome to promote ribosome biogenesis (*15*). Mutations in DKC1 [nucleolar protein at 60B (Nop60B) in *Drosophila*] can also impair ribosome binding to transfer RNAs (tRNAs) and to internal ribosomal entry sites from yeast to humans (*16*). Nevertheless, how H/ACA snRNP complex dysfunction generates tissue-specific defects remains unclear.

**Fig. 1.**
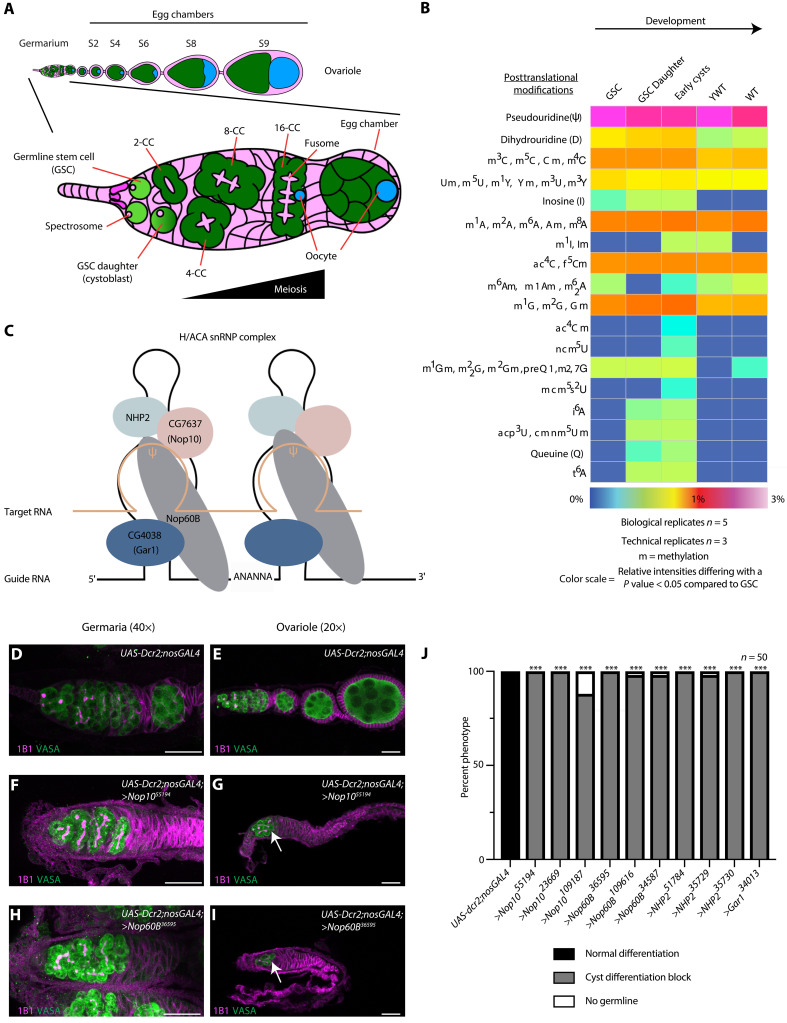
Pseudouridine is a critical modification required for oogenesis. (**A**) Schematic of *Drosophila* ovariole (top) and germarium (bottom). (**B**) Heatmap of mass spectrometry analysis of RNA modifications at each developmental stage (as described in Materials and Methods). A cold-hot gradient covers relative abundances from 0 to 3%. The different colors also express variations of relative abundance from the GSC column with a *P* < 0.05 statistical significance. (**C**) Schematic of the H/ACA snRNP complex comprising CG4038 (Gar1) (dark blue), Nop60B (gray), NHP2 (light blue), and CG7637 (Nop10) (salmon). (**D** to **I**) Confocal image of germarium of *UAS-Dcr2;nosGAL4* (D and E), *Nop10* germline depletion (F and G), and *Nop60B* germline depletion (H and I) stained with anti1B1 (magenta) and anti-Vasa (green) antibodies. Images were taken at either 40× (germaria) or 20× (ovariole). The white arrow marks cyst defect. Scale bars, 20 μm. (**J**) Quantification of oogenesis defect phenotypes. Statistical analysis performed with Fisher’s exact test (*n* = 50, ***P < 0.0001).

During *Drosophila* oogenesis, differentiation of a germline stem cell (GSC) to an oocyte is sensitive to both ribosome biogenesis and translation (*1*, *6*, *8*, *9*, *17*). Oogenesis occurs in ovarioles, beginning with the GSCs in the germaria (Fig. 1A). The GSCs undergo asymmetric cell division to self-renew and give rise to daughter cells called cystoblasts (CBs) (*18*, *19*). During CB differentiation, the CB undergoes four incomplete mitotic divisions giving rise successively to 2-, 4-, 8-, and, finally, 16-cell cysts (*20*). One cell in the 16-cell cyst stage becomes the oocyte, while the remaining 15 cells become nurse cells that support the growing oocyte (Fig. 1A) (*21*). The GSCs and the CBs are marked by a round cytoskeletal structure called the spectrosome, while the cysts are marked by a branched structure called the fusome (*22*). The 16-cell cyst becomes encapsulated by somatic cells to create an egg chamber that then goes through progressive development to produce a mature egg (Fig. 1A) (*21*).

In the CB, expression of Bag of marbles (Bam) promotes the progression from CB to an 8-cell cyst stage where expression of RNA-binding Fox protein 1 (Rbfox1) and Bruno 1 (Bru1) are required to specify an oocyte (*23*, *24*). In parallel, several cells in the cysts initiate recombination that is mediated by the synaptonemal complex, which includes proteins such as crossover suppressor on 3 of Gowen [c(3)G], but only the specified oocyte commits to meiosis (*25*, *26*). Rbfox1 is critical not only for female fertility but also for neurological functions and is sensitive to levels of ribosomes during *Drosophila* oogenesis (*23*, *27*, *28*). Why some transcripts encoding differentiation factors, such as Rbfox1, are sensitive to ribosome levels is not known.

## RESULTS

### RNA modifications are dynamic and essential for oogenesis

We aimed to identify dynamic RNA PTMs during oogenesis. Therefore, we enriched for five stages of oogenesis {1, GSCs; 2, GSC daughter/CBs; 3, early cysts; 4, germaria and early-stage egg chambers [young wild type (YWT)]; and 5, late-stage egg chambers [wild type (WT)]}, which are critical milestones of germline development (*19*, *29*, *30*). We performed tandem mass spectrometry on total RNA extracted from each of the enriched stages (fig. S1, A and B) (*19*, *29*, *30*). For each enriched developmental stage, we performed five biological replicates, each with three technical replicates. We identified 18 groups of RNA PTMs represented by distinct mass-to-charge ratios, composed of 42 distinct RNA PTMs from a total of 172 known PTMs (Fig. 1B and table S1). Pseudouridine, which is the most frequent PTM in RNA (*31*), was the most abundant modification at all stages, followed by the monomethylations of the canonical RNA bases (Fig. 1B and table S1). Furthermore, we found a cohort of RNA PTMs, including inosine and dihydrouridine, that were not previously described during oogenesis (Fig. 1B and table S1). Most RNA PTMs, including pseudouridine, were dynamic during GSC differentiation into an oocyte (Fig. 1B and table S1).

To determine whether the RNA modifications play a role in germline development, we performed an RNA interference (RNAi) screen using a germline-specific *nanos-GAL4* driver and *UAS-RNAi* lines to deplete RNA-modifying enzymes in the germline, followed by immunostaining for Vasa, a germline marker, and 1B1, a marker of the somatic cell membranes, spectrosomes, and fusomes (*32*, *33*). We screened 33 unique genes annotated and predicted to be involved in RNA modification, using more than one independent *UAS-RNAi* line when available for a total of 48 lines. Of the 33 distinct gene knockdowns, 2 resulted in loss of the germline, 14 in germarium defects, and 3 in egg chamber defects (table S2).

### The pseudouridine-depositing H/ACA snRNP complex is required for oocyte specification

Among the genes whose knockdown caused defects in germaria, we found all four encoding components of the rRNA pseudouridine synthase, the H/ACA snRNP complex: the catalytic subunit Nop60B and complex members CG7637 (Nop10), CG4038 (Gar1), and NHP2 (Fig. 1C and table S2). Germline depletion of Nop60B in the background of a green fluorescent protein (GFP)–tagged Nop60B under endogenous control led to significantly reduced GFP levels in the germline (fig. S2, A to C), verifying knockdown of *Nop60B*. In addition, reverse transcription quantitative polymerase chain reaction analysis revealed significantly reduced levels of *Nop10* and *Nop60B* mRNAs upon germline knockdown (fig. S2D). Depletion of the H/ACA snRNP complex components did not result in a germline viability defect but rather a specific loss of GSCs and a cyst differentiation defect. Previously, it has been shown that reduced ribosome biogenesis results in the loss of GSCs and a GSC abscission defect rather than a cyst differentiation defect (*6*, *9*). Here, we focus on the cyst differentiation defect that results upon depletion of the H/ACA snRNP complex. Specifically, transition from the 8-cell cyst stage to an egg chamber was blocked, as measured by the accumulation of 8-cell cysts (Fig. 1, D to J; fig. S2, E to O; and table S2) (*6*, *34*), which led to an absence of egg chambers and, in turn, sterility (fig. S2P). We found that depletion of Nop60B resulted in a severe defect compared to other members of the H/ACA snRNP complex. This could be due to either Nop60B being the catalytic subunit of the H/ACA snRNP or the efficiency of RNAi. Thus, the H/ACA snRNP complex is required in the female germline for proper cyst differentiation.

**Fig. 2.**
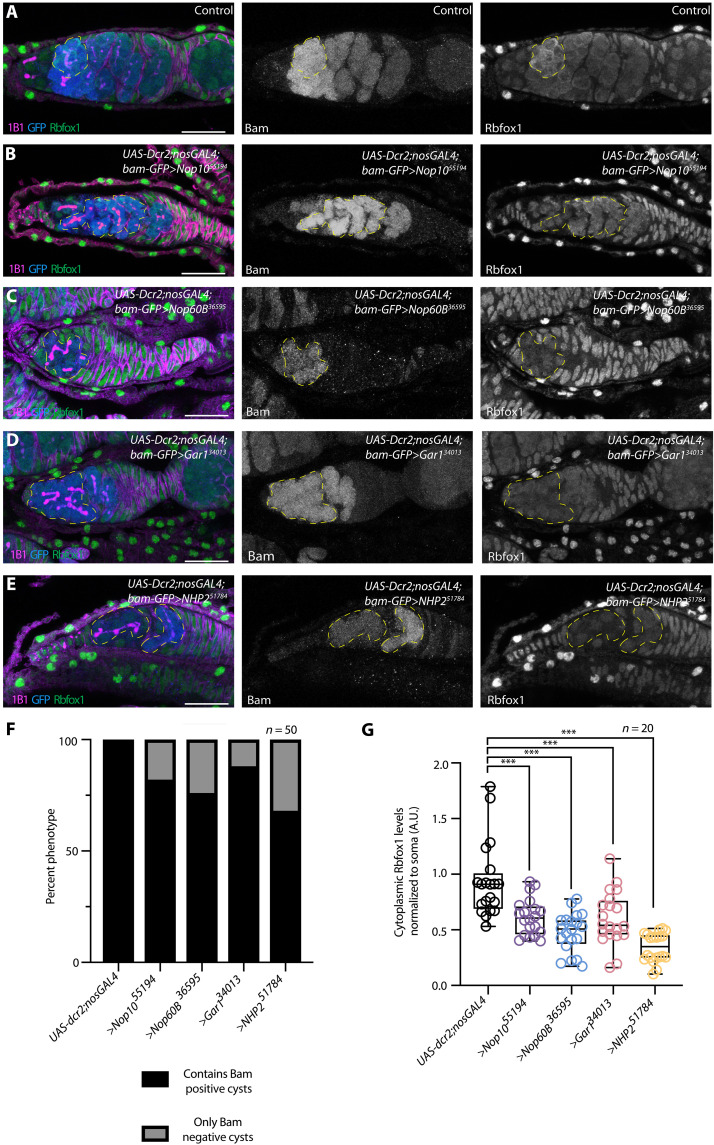
The H/ACA snRNP complex is required for proper cyst differentiation and meiotic progression. (**A** to **E**) *UAS-Dcr2;nosGAL4;bam-GFP* (A) and germline depletion of *Nop10* (B), *Nop60B* (C), *Gar1* (D), and *NHP2* (E) stained with anti-1B1 (magenta), anti-GFP (blue), and anti-Rbfox1 (green). GFP and Rbfox1 are shown in gray scale. Yellow dashed lines outline cysts that are positive for GFP but have lower Rbfox1 levels for all images. Scale bars, 20 μm. (**F**) Quantification of oogenesis defect phenotypes per genotype. Statistical analysis performed with Fisher’s exact test (*n* = 50, ***P < 0.0001). (**G**) Quantification of Rbfox1 levels normalized to soma for *Nop10*, *Nop60B*, *Gar1*, or *NHP2* germline depletion. Statistics were performed using Dunnett’s multiple comparisons test and post hoc test after one-way analysis of variance (ANOVA) (*n* = 20, ***P < 0.0001). A.U., arbitrary units.

We further investigated the role of the H/ACA snRNP complex in cyst differentiation by analyzing control and H/ACA snRNP complex germline–depleted ovaries carrying the differentiation reporter, BamGFP. We also stained ovaries for Vasa, 1B1, and the cyst-differentiation factors, Rbfox1 or Bru1 (*18*, *23*, *24*). We found that cysts lacking H/ACA snRNP complex members expressed BamGFP but had significantly reduced levels of Rbfox1 and Bru1 (Fig. 2, A to G, and fig. S3, A to D). Moreover, cysts lacking H/ACA snRNP complex components did not specify an oocyte, as cysts were devoid of localized Egalitarian (Egl), the oocyte determinant, and exhibited reduced expression of the synaptonemal complex component C(3)G (fig. S3, E to L) (*26*, *35*, *36*), consistent with a differentiation block.

To determine the specific stage of oogenesis that requires H/ACA snRNP complex activity, we first characterized the expression of Nop60B::GFP. Nop60B::GFP levels increased from the cyst stages to early egg chambers (fig. S4, A and B). Using a pseudouridine antibody, we observed a corresponding increase in pseudouridine levels from the 8-cell cyst to the newest egg chamber, and this increase depended on the H/ACA snRNP complex (fig. S4, C to G). Given these observations and that loss of H/ACA snRNP complex components resulted in an accumulation of 8-cell cysts (fig. S2O) (*34*), we hypothesized that the H/ACA snRNP complex is required in the cysts for the transition into an oocyte. To test this, we depleted *Nop60B* and *Nop10* in the cysts by RNAi using a *bamGAL4* driver, which is active in the 2- to 8-cell cyst stages. We observed an accumulation of cysts with significantly reduced levels of Rbfox1 (fig. S5, A to H) (*18*, *23*). Together, these data suggest that the H/ACA snRNP complex is required in the cyst stages for the final mitotic division to produce a 16-cell cyst and for differentiation into an oocyte.

### The H/ACA snRNP complex promotes ribosome biogenesis and the translation of differentiation factors during oogenesis

**Fig. 3.**
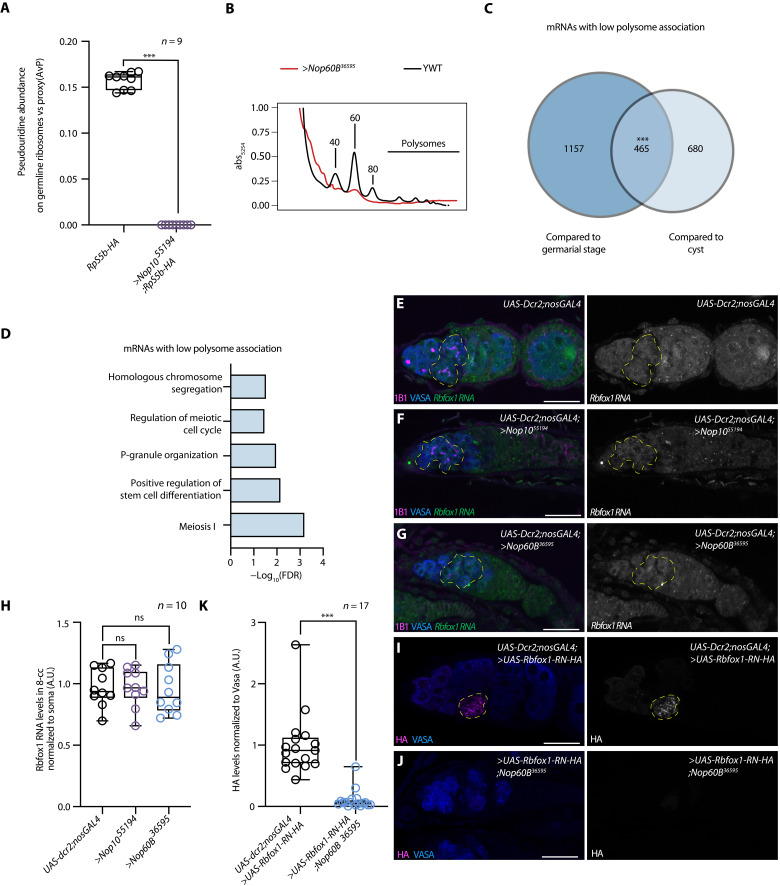
The H/ACA snRNP complex is required for translation of meiotic mRNAs. (**A**) Mass spectrometry analysis of rRNA from germline ribosomal pulldowns. Statistics was performed using *t* test. For each genotype, at least two biological replicates were analyzed, with three technical replicates each (***P < 0.0001). (**B**) Polysome traces for YWT (*UAS-Dcr2;nosGAL4*) (black) and *Nop60B* germline depletion (red). Nop60B is required for proper ribosome biogenesis as loss of Nop60B results in 40*S* and 60*S* defects and reduction of polysomes compared to control. (**C**) Venn diagram illustrating overlap of Nop60B-polysome ≤2-fold less association with the ribosome (*n* = 2, *e* < 2.87 × 10^−192^, hypergeometric test). Germarial stages consist of ovaries from young *UAS-Dcr2;nosGAL4*, while the cyst stages are from *bam RNAi; hs-bam* (enrichment described in Materials and Methods). (**D**) Significant biological process GO terms of shared lowly associated mRNAs in *Nop60B*. (**E** to **G**) In situ hybridization to *Rbfox1* RNA (green/gray) and staining with anti-1B1 (magenta) and anti-Vasa (blue) antibodies in *UAS-Dcr2;nosGAL4* (E) and *Nop10* germline depletion(F) or *Nop60B* germline depletion(G). Yellow dashed lines outline *Rbfox1* RNA. (**H**) *Rbfox1* RNA levels in *Nop10* and *Nop60B* germline depletions normalized to soma. Statistics were performed using Dunnett’s multiple comparisons test and post hoc test after one-way ANOVA (*n* = 10, ns, *P* > 0.9999 and *P* = 0.9792, respectively). (**I** and **J**) Germarium of *UAS-Dcr2;nosGAL4* driving *UAS-Rbfox1-RN-HA* (I) and UAS-Dcr2;nosGAL4 driving *Rbfox1-RN-HA* in the background of *Nop60B* germline depletion (J). Germaria stained with anti-Vasa (blue) and anti-HA (magenta) antibodies. (**K**) HA levels in control and *Nop60B* germline depletion normalized to Vasa. Statistics were performed using unpaired *t* test (*n* = 17, ***P < 0.0001). Yellow dashed lines outline Rbfox1-RN-HA.

The primary activity of the H/ACA snRNP complex is to deposit pseudouridine on rRNA, thereby promoting ribosome biogenesis in the nucleolus (*37*). Nop60B::GFP colocalized with fibrillarin in the nucleolus as previously observed (fig. S6, A and B) (*38*). In addition, loss of Nop10 and Nop60B resulted in cysts with hypertrophic nucleoli as compared to WT cysts, suggesting a ribosome biogenesis defect (fig. S6, C to F) (*6*, *10*). Loss of DKC1, dyskerin pseudouridine synthase 1, or Nop60B in S2 cells resulted in the accumulation of rRNA intermediates, suggesting that the H/ACA snRNP complex plays a role in rRNA maturation and ribosome biogenesis (*6*). To verify that the H/ACA snRNP complex deposits pseudouridine on rRNA during oogenesis, we coimmunopurified the 40*S* and 60*S* ribosomal subunits from the germline, using a germline-enriched hemagglutinin (HA)–tagged ribosomal protein RpS5b (fig. S6, G to I) (*39*, *40*). Mass spectrometry analysis showed that loss of the H/ACA snRNP complex member Nop10 led to a significant decrease of pseudouridine on rRNA relative to controls (Fig. 3A and table S3). In addition, we observed a decrease in both the 40*S* and 60*S* subunits and in polysomes of Nop60B-depleted ovaries as compared to controls (Fig. 3B) (*41*), suggesting a ribosome biogenesis defect upon loss of the H/ACA snRNP complex. Thus, consistent with previous findings, the H/ACA snRNP complex deposits pseudouridine on rRNA to promote ribosome biogenesis in the germline.

To test whether loss of the H/ACA snRNP complex and consequent aberrant ribosome biogenesis affects mRNA translation during oogenesis, we performed polysome sequencing of ovaries depleted of Nop60B in the germline and of gonads enriched for cyst stages (*9*, *19*, *29*). As enriching for early cyst stages includes a heat shock step (see Materials and Methods), we also analyzed early-stage egg chambers to control for heat shock effects. We detected 465 mRNAs with a reduced polysome association in *Nop60B*-knockdown versus the controls, whereas 638 mRNAs showed an increased polysome association (Fig. 3C and fig. S7, A to C). These data suggest that the H/ACA snRNP complex regulates the synthesis of a cohort of proteins. Gene Ontology (GO) term analysis revealed that mRNAs with an elevated polysome association coded for proteins that promote mitotic cell cycle, whereas those with reduced polysome association coded for proteins that promote meiosis 1, meiotic cell cycle, and homologous chromosome segregation (Fig. 3D and fig. S7D), such as the synaptonemal complex members C(3)G and Corona (Cona), consistent with reduced C(3)G protein levels upon depletion of the H/ACA snRNP complex (fig. S3, I to L).

The levels of *Rbfox1* and *Bru1* mRNAs were not significantly reduced in the germline upon depletion of the H/ACA snRNP complex, as indicated by fluorescence in situ hybridization (Fig. 3, E to H, and fig. S7, E to K). To determine whether the H/ACA snRNP complex is required for translation of *Rbfox1* and *Bru1*, we used published fly lines that express the CDS of either Rbfox1 or Bru1 under control of the Upstream Activation Sequence (UAS)/GAL4 system (*23*, *42*). These proteins were detected in the control germaria but not in the H/ACA snRNP complex–depleted germaria (Fig. 3, I to K, and fig. S7, L to N), suggesting that their translation is impaired upon loss of the H/ACA snRNP complex.

We considered that the H/ACA snRNP complex is required for oogenesis due to its role in ribosome biogenesis. This hypothesis predicts that compromised ribosome biogenesis will phenocopy loss of H/ACA snRNP complex components. We impaired ribosome biogenesis by depleting ribosomal protein paralogs RpS10b and RpS19b in the germline, as the depletion of other ribosomal proteins that do not have paralogs results in GSC differentiation defects or loss of cyst stages that would mask the cyst differentiation block (*6*, *39*, *43*, *44*). Depletion of RpS10b and RpS19b phenocopied loss of H/ACA snRNP complex components, leading to a block in cyst differentiation and decreased levels of Rbfox1 and Bru1 proteins without a concomitant loss of their mRNAs (figs. S8, A to K, and S9, A to H). The H/ACA snRNP complex can also pseudouridylate mRNAs and tRNAs. However, immunopurification of pseudouridine did not enrich for the mRNAs with perturbed translation upon loss of the H/ACA snRNP complex (table S4), suggesting that these targets are not pseudouridylated. In addition, whereas the loss of the H/ACA snRNP complex blocked cyst differentiation, loss of tRNA pseudouridylation enzymes resulted in a different phenotype—loss of cyst stages (fig. S10, A to J). Thus, our data suggest that the H/ACA snRNP complex and pseudouridinylation are important for ribosome biogenesis during oogenesis.

### H/ACA snRNP complex–dependent differentiation factors are polyQ proteins

**Fig. 4.**
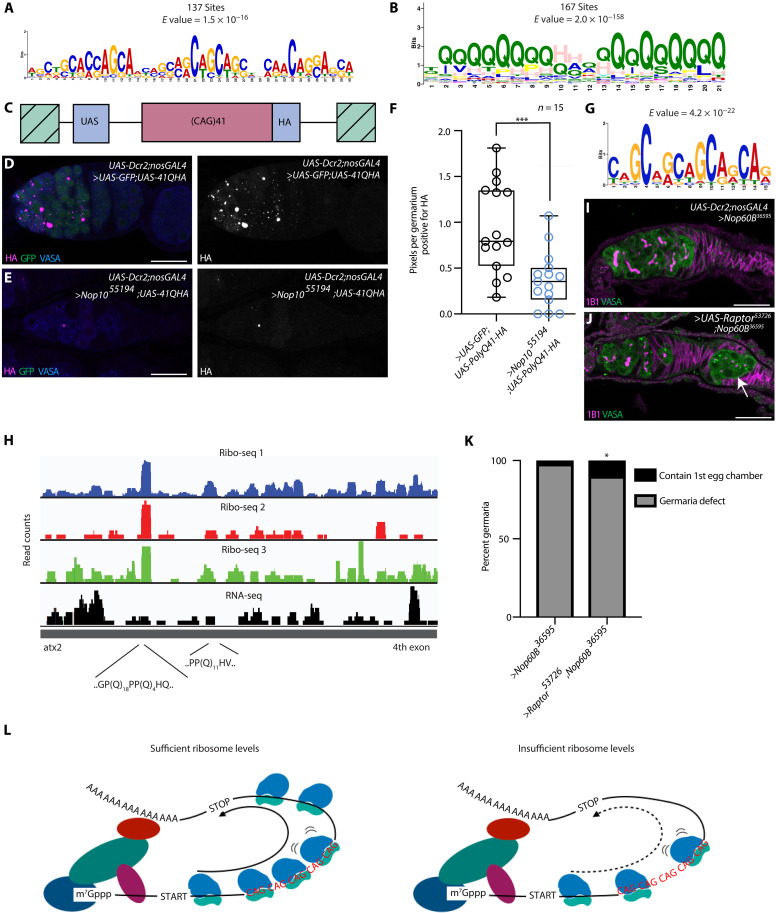
The H/ACA snRNP complex is required for translating polyQ proteins. (**A**) CAG motif identified by MEME enriched in the CDS of mRNAs lowly associated with polysomes. (**B**) PolyQ motif identified by MEME that is enriched in the amino acid sequence of mRNAs lowly associated with the ribosome. (**C**) Schematic of the CAG reporter used. (**D** and **E**) *UAS-poly41Q-HA* driven in *UAS-Dcr2;nosGAL4* (D) and in the backgorund of *Nop10* germline depletion (E) stained with anti-HA (magenta/gray), anti-GFP (green), and anti-Vasa (blue) antibodies. Scale bar, 20 μm. (**F**) HA reporter quantification using unpaired *t* test (*n* = 15, ***P = 0.0007). (**G**) CAG motif identified by MEME detected by ribosome footprinting. (**H**) Ribosome footprint distribution on *atx2* mRNA. (**I** and **J**) *Nop60B* (I) and *Nop60B* rescue by overexpression of the Tor pathway member Raptor (J) stained with anti-Vasa (green) and anti-1B1 (magenta). The arrow points at the egg chamber. (**K**) Quantification of *Nop60B* depletion phenotypic rescue by overexpression of the Tor pathway member Raptor in H/ACA box knockdown (>Nop60B RNAi, *n* = 91, 1.1% contained the first egg chamber, while for *UAS-Nop60B RNAi;UAS-Raptor*, *n* = 151, 9.9% contained the first egg chamber, Fisher’s exact test, *P = 0.0037). (**L**) Representative model showing that a sufficient level of ribosomes is required for translation of meiotic CAG containing mRNAs promoting terminal differentiation. Ribosome insufficiency reduces translation of meiotic CAG containing mRNAs, due to ribosome stalling or slowing, causing terminal differentiation failure.

To identify shared properties among the mRNAs with reduced polysome association upon loss of the H/ACA snRNP complex, we performed a motif analysis of the 5′ untranslated regions (5′UTRs), coding sequence (CDS), and 3′UTRs of this subset of mRNAs compared to a set of control mRNAs. We observed a motif of repeating CAG nucleotides that was highly enriched in the CDS of downregulated transcripts compared to the control unregulated mRNAs (Fig. 4A). In addition, we found motifs that were enriched in the 3′UTRs or 5′UTRs of down-regulated transcripts, albeit in a smaller subset of RNAs (table S5). The CAG motifs in the down-regulated transcripts were in-frame, such that the encoded proteins are highly enriched in glutamine (Q) over a region of 21 amino acids (Fig. 4B). *Rbfox1* and *Bru1* both contain a region that consists of repeating CAG motifs of different lengths in the mRNA and polyQ in the protein, respectively (fig. S11, A to D) (*27*).

To determine whether the H/ACA snRNP complex is required to translate CAG repeat–containing mRNAs during oogenesis, we expressed a CAG reporter encoding an HA-tagged polyQ protein, which was previously used to model polyglutamine toxicity in Huntington’s disease (Fig. 4C) (*45*). We coexpressed the CAG reporter with GFP in the control, to ensure equal GAL4 dosage, and the CAG reporter in Nop10-depleted germlines. We found that loss of the H/ACA snRNP complex specifically reduced the levels of the polyQ protein (Fig. 4, D to F, and fig. S11, E and F). Furthermore, depletion of RpS19b and Nop60B also resulted in a significant decrease in polyQ protein accumulation, but loss of Nop60B did not significantly alter the levels of GFP (fig. S11, G to M). Thus, the H/ACA snRNP complex and ribosome biogenesis are required for translation of polyQ-containing proteins.

### CAG repeat regions show increased density of ribosomes

One proposed mechanism of polyQ expansion-induced defects is the disruption of translation by ribosome stalling (*46*). To determine whether the H/ACA snRNP complex affects elongating ribosomes on, and hence translation of, mRNAs encoding polyQ proteins, we performed ribosome footprinting (Ribo-Seq). Because we were unable to acquire sufficient material from H/ACA snRNP complex–depleted germaria, we used ovaries enriched for late-stage oocytes (late ovaries), which have reduced pseudouridine levels but can be obtained in sufficient quantity (Fig. 1B and table S1). Three late-ovary Ribo-Seq libraries were generated, each with a corresponding RNA-sequencing (RNA-seq) library. Correlation analysis showed consistent and reproducible ribosome footprint distributions among the three Ribo-Seq libraries (Pearson *r* > 0.9 for all comparisons; table S6). We hypothesized that stalled ribosomes might result in local enrichment of ribosome footprints. We therefore sought to identify peaks in the Ribo-Seq data across the transcriptome. Our peak detection and subsequent motif analysis identified 123 mRNAs containing at least one CAG-rich segment within 30 nucleotides of a ribosome footprint peak in at least two of the three Ribo-Seq libraries that were not present in the RNA-seq libraries (Fig. 4G and table S6). Forty-six of the 123 identified mRNAs encode at least one polyQ tract (≥4 consecutive CAG codons) near ribosome footprint peaks (Fig. 4H and table S6). Cytoplasmic Rbfox1 that contains the polyQ motif was not identified as it is not expressed in the later stages (*23*). Furthermore, 45 of 50 transcripts were found to have more than 20% increase in ribosome occupancy within polyQ, polyS, and polyA regions, while 34 of 50 had more than twofold increase (fig. S12A). This motif is highly reminiscent of the motif identified in the mRNAs with low polysome association in H/ACA snRNP complex depleted germlines (Fig. 4A).

The motif identified by Ribo-Seq contains five CAGs in a row (Fig. 4, A and 4G). To determine whether the 5-CAG motif is overrepresented in the set of target mRNAs with low polysome association, we performed a Find Individual Motif Occurrence (FIMO) analysis. We found that 181 of 465 (39%) targets contain a significant motif representative of the 5-CAG motif including *bru1* and *C(3)G*, which is greater than the proportion of 5-CAG motif seen in all highly expressed mRNAs in the cyst stages (~21.5%) (table S7). We infer that CAG repeat sequences have high ribosome density and are present in mRNAs whose translation is sensitive to reduced ribosome biogenesis during oogenesis.

### Tor signaling partially restores differentiation and modulates polyQ translation

The Tor pathway is a critical positive regulator of ribosome biogenesis. To further determine whether loss of the H/ACA snRNP complex blocks cyst differentiation due to reduced ribosome biogenesis, we increased ribosome biogenesis by overexpressing the Target of Rapamycin Complex 1 (TORC1) cofactor, Raptor, in the H/ACA snRNP complex–depleted germline (*47*). We observed a partial yet significant alleviation of the cyst differentiation defect, such that an egg chamber was formed (Fig. 4, I to K). We next asked whether decreasing TOR activity and, in turn, ribosome biogenesis could diminish expression of the polyQ reporter. Specifically, flies expressing the reporter were treated with the inhibitor of mammalian target of rapamycin (mTOR), and displayed lower levels of germline polyQ, a loss of germline, and lower Rbfox1 protein levels when compared to controls (fig. S13, A to G). Furthermore, our FIMO analysis identified that Aramis contains a polyQ motif (table S7). Using a tagged Aramis-GFP, we found that loss of H/ACA snRNP resulted in a reduction in Aramis-GFP, and overexpression of Raptor partially rescued Aramis-GFP expression (fig. S13, H to K). Thus, our data suggest that modulating ribosome levels via the Tor pathway can effectively regulate translation of polyQ-containing proteins.

## DISCUSSION

We used the power of *Drosophila* genetics in combination with mass spectrometry to determine the developmental profile of RNA PTMs and identify a cohort of PTMs that are required for proper oogenesis. Specifically, we found that pseudouridine abundance is dynamic and regulated by the H/ACA snRNP complex, a pseudouridine synthase, and is required for proper cyst differentiation and oocyte specification. Using polysome-sequencing (polysome-seq) analysis, we found that CAG repeat mRNAs encoding polyQ-containing proteins have reduced polysome association upon loss of the H/ACA snRNP complex. These polyQ proteins include germ cell differentiation and meiosis promoting factors such as Rbfox1 and Bru1. Moreover, we found that CAG repeat regions accumulate ribosomes, potentially acting as a ribosome sink. Together, our data suggest that under the condition of low ribosome levels, the CAG repeat containing regions can impede proper translation by sequestering ribosomes internally, thereby causing translation of polyQ-containing proteins to be sensitive to ribosome levels (Fig. 4L). Together, we find that the H/ACA snRNP complex promotes ribosome biogenesis during oogenesis and, in turn, the translation of CAG repeat mRNAs required for differentiation (Fig. 4L).

Ribosomopathies predispose individuals to neurological deficits, but the etiology of this defect is unclear (*41*, *48*). Neurons express and require several polyQ-containing proteins, including Rbfox1 (*27*, *28*, *49*). We find that the translation and levels of Rbfox1 are sensitive to ribosome levels during oogenesis (*44*). By extension, neuronal deficits observed in ribosomopathies could be due to inability to translate critical polyQ-containing proteins in neurons.

While polyQ stretches facilitate phase transition, large CAG expansion and polyQ protein aggregates are associated with diseases such as Huntington’s disease (*50*). A genome-wide association study revealed that the onset of Huntington’s disease is due to large expansions of CAG repeats and is accelerated by DNA repair genes as well as E3 ubiquitin protein ligase (UBR5) (*51*). In embryonic stem cells, UBR5 has been shown to physically interact with the H/ACA snRNP complex to promote rRNA maturation, suggesting that these factors could collaborate to promote early onset of Huntington’s disease (*52*). Furthermore, Huntington’s disease mouse models have shown that CAG expansions induce ribosome stalling by impeding ribosome translocation, thereby inhibiting protein synthesis (*46*). These data, together with our findings that during development the H/ACA snRNP complex promotes translation of CAG repeat containing RNAs, suggest that translation dysregulation could be a key feature of CAG expansion diseases. While the early onset is determined by CAG length, translation of CAG repeats into polyQ proteins can cause protein aggregation and toxicity (*51*). Our finding that translation of such polyQ proteins is sensitive to ribosome levels reveals new potential therapeutic targets. For instance, several TOR inhibitors have been generated to primarily treat cancers; the mechanism we have identified provides a potential pathway to repurpose these drugs to reduce polyQ protein aggregation in various repeat expansion disease states (*53*–*55*).

## MATERIALS AND METHODS

### Fly lines

Flies were grown at 25° to 29°C and dissected between 0 to 3 hours or 1 to 3 days after eclosion. Heat shock experiments were performed on 1-day-old flies. The following RNAi lines were used in this study: *nsun2* RNAi (Bloomington, #62495), *Trm7-34* RNAi (Bloomington, #62499), *CG32281* RNAi (Bloomington, #51764), *rswl* (Bloomington, #44494), *CG9386* (Bloomington, #33364), *Nsun5* (Bloomington, #32400), *CG11109* (Bloomington, #56897), *CG11447* (Bloomington, #43207), *CG3021* (Bloomington, #55144), *RluA-2* [Vienna Drosophila Resource Center(VDRC), #34152], *RluA-2* (VDRC, #106382), *Wuho* (Bloomington, #61281), *CG10903* (VDRC, #57481), *RluA-1* (VDRC, #41757), *RluA-1* (VDRC, #41758), *RluA-1* (VDRC, #109586), *Tailor* (Bloomington, #36896), *Pus1* (Bloomington, #53288), *NOP60B* (Bloomington, #36595), *CG6745* (Bloomington, #41825), *CG7637* (Bloomington, #55194), *NHP2* (Bloomington, #51784), *CG4038* (Bloomington, #34013), *CG34140* (Bloomington, #38951), *CG34140* (Bloomington, #57311), *CG3645* (VDRC, #107156), *CG1434* (VDRC, #104876), *CG3434* (VDRC, #45130), *tgt* (VDRC, #41644), *AlkB* (Bloomington, #43300), *Paics* (Bloomington, #62241), *Ras* (Bloomington, #31654), *Ras* (Bloomington, #51717), *Ras* (Bloomington, #31653), *pfas* (Bloomington, #36686), *pfas* (Bloomington, #80831), *bam* RNAi;*hs-bam* (B. Ohlstein and D. McKearin), *RpS10b* (Bloomington, #43976), *RpS19b* (VDRC, #22073), *UAS-raptor-HA* (Bloomington, #53726), *Aramis::GFP* (VDRC, #318713), and *Nop60B::GFP* (VDRC, 318245).

The following tissue-specific drivers were used in this study: *UAS-Dcr2*;*nosGAL4* (Bloomington, #25751), *UAS-Dcr2*;*nosGAL4*;*bam-GFP* (Lehmann laboratory), *If*/*CyO*;*nosGAL4* (Lehmann laboratory), *nosGAL4*;*MKRS*/*TM6* (Bloomington, #4442), and *TjGAL4*/*CyO* (Lehmann laboratory). Other fly lines used in this study were as follows: *RpS5b-HA* and *UAS-Rbox1-RN* (Buszczak laboratory) and *UAS-Tkv* (Bloomington, #36536), *UAS-Rbox1-RN* (Buszczak laboratory), *UAS-Bruno* (Ephrussi laboratory), and *UAS-41Q.HA* (Bloomington, #30540).

### Rapamycin treatment

One day before treatment, 400 μl of 100 μM rapamycin or 400 μl of ethanol was added to the top of food and allowed to dry. Flies were crossed at 18°C and collected 1 to 2 days after eclosion. Flies were placed on food and temperature shifted to 29°C. Every other day, flies were placed onto fresh food with 400 μl of 100 μM rapamycin or 400 μl of ethanol for a total of 7 days. Flies were dissected as described below.

For treatment to analyze Rbfox1 expression, 1 day before treatment, 500 μl of 100 μM rapamycin or 500 μl of ethanol was added to the top of food and allowed to dry. Flies were placed on food at 25°C. Every other day flies were placed onto fresh food with 500 μl of 100 μM rapamycin or 500 μl of ethanol for a total of 7 days. Flies were dissected as described below. Note that variations in Rbfox1 expression were observed with rapamycin treatment.

### Genotypes used to enrich specific stages of germline and fragment analyzer

To enrich for GSCs: *nosGAL4>UAS-tkv*. Cystoblasts: *nosGAL4>bam RNAi*. Differentiating cysts: *nosGAL4>bam RNAi;hs-bam *(*30*, *44*). Female flies were heat-shocked at 37°C for 2 hours, incubated at room temperature for 4 hours, and heat-shocked again for 2 hours. This was subsequently repeated the next day, and flies were dissected. YWT: Female flies were collected and dissected within 2 hours of eclosion. To dissect WT ovaries, 2- to 3-day-old females (*UAS-Dcr2;nosGAL4*) were fattened overnight and dissected the next day. In addition, to analyze H/ACA snRNP total RNA, *UAS-Dcr2;nosGAL4* and >*Nop60B^36595^* genotypes were used.

To analyze relative RNA contributions, total RNA was extracted from the above genotypes. Total RNA was subjected to turbo deoxyribonuclease (DNase) treatment as described in the RNA isolation protocol. Either about 40 or 120 ng of total RNA, depending on experiment (see figure legends), was subjected into an Agilent Technologies CE System 5200 Fragment Analyzer using the DNF-471-0500 kit as per the manufacturer’s protocol.

### Dissection and immunostaining

Ovaries were dissected in phosphate-buffered saline (PBS) and fixed for 10 min in 5% methanol-free formaldehyde (*44*). Samples were washed in 1 ml of PBT (PBS, 0.5% Triton X-100, and 0.3% bovine serum albumin) four times for 10 min each. Primary antibodies were added in PBT and incubated at 4°C rotating overnight. Samples were washed three times for 10 min each in 1 ml of PBT and once in 1 ml of PBT with 2% donkey serum (Sigma-Aldrich) for 10 min. Secondary antibodies were added in PBT with 4% donkey serum and incubated at 4°C rotating overnight. Samples were washed four times for 10 min each in 1 ml of PBST (0.2% Tween-20 in PBS). Vectashield with 4′,6-diamidino-2-phenylindole (Vector Laboratories) was added for 30 min before mounting. The following primary antibodies were used: mouse anti-1B1 (1:20; Developmental Studies Hybridoma Bank (DSHB), rabbit anti-Vasa (1:1000; Rangan laboratory), chicken anti-Vasa (1:1000), rabbit anti-GFP (1:2000; Abcam, ab6556), rabbit anti-Egl (1:1000; Lehmann laboratory), mouse anti-pseudouridine (1:1000; MBL Life Sciences), mouse anti-C3G (1:1000; Hawley laboratory), rat anti-HA(1:500; Roche, 11 867 423 001), mouse anti-fibrillarin (1:50; Fuchs laboratory), guinea pig anti-Rbfox1 (1:1000; Buszczak laboratory), and rabbit anti–phosphorylated-S6 (1:200; Teleman laboratory).The following secondary antibodies were used: Alexa Fluor 488 (Molecular Probes), Cy3, and Cy5 (the Jackson Laboratory) were used at a dilution of 1:500.

### Fluorescence in situ hybridization

Probes were designed and generated by LGC Biosearch Technologies using Stellaris RNA FISH Probe Designer, with specificity to target base pairs of target mRNAs. Ovaries (three pairs per sample) were dissected in ribonuclease (RNase)–free PBS and fixed as described above. The fixed tissue was washed twice with 1 ml of PBS and then permeabilized with 70% ethanol at 4°C for 2 hours. After permeabilization, 1 ml of wash buffer was added (40 ml of RNase-free water, 5 ml of deionized formamide, and 5 ml of 20× SSC) for a 5-min wash. To the sample, 50 μl of a Stellaris hybridization buffer, 10% (v/v) of formamide with 50 to 100 mM of oligos, and diluted antibodies were added and incubated at 30°C for a minimum of 16 hours in the dark. After the overnight incubation, the sample was washed twice with 1 ml of wash buffer with properly diluted secondary antibodies for 30 min. After the second wash, Vectashield was added, and samples were imaged.

Stellaris probes were designed (https://biosearchtech.com/support/education/stellaris-rna-fish) for all possible isoforms. Sequences are found in Excel file primerlist.xslx.

### Fluorescence imaging

Ovaries were visualized under 20× oil and 40× oil objective lenses, and images were acquired using a Zeiss LSM 710 confocal microscope. Confocal images were processed with ImageJ. The images were quantified using ImageJ with the Measurement function.

### AU quantification of protein or in situ

To quantify immunofluorescence intensities for Rbfox1, Bruno, GFP, and pseudouridine or in situ hybridization probe fluorescence in germ cells, images for both control and experimental germaria were taken using the same confocal settings. Z stacks were obtained for all images. Similar planes in control and experimental germaria were chosen, and the area of germ cells positive for the proteins or RNAs of interest was outlined and analyzed using the “analyze” tool in Fiji (ImageJ). The mean intensity and area of the specified region were obtained. An average of all the ratios (mean/area), for the proteins or RNAs of interest, per image was calculated for both control and experimental conditions. Germline intensities were normalized to somatic intensities or if the protein or RNA of interest is germline-enriched and not expressed in the soma, they were normalized to Vasa or background. The highest mean intensity between control and experimental(s) was used to normalize to a value of 1 arbitrary unit on the graph.

To quantify polyQ-HA, images were first filtered with a median pixel of 1. The program set the threshold values using max entropy threshold for the images, and the outline of the germline was traced using the germline marker Vasa. The percent pixel count per the germline area was found and normalized to the highest mean intensity between control and experimental(s). For rapamycin treatment, 15 control and 15 treated germaria were used. Three randomly selected slices of each stack (total of 45 slices) were quantitated for both control and rapamycin treated germaria.

### Egg-laying assay

Egg-laying assays were conducted in triplicate in vials containing standard fly food. Before the assay, dry yeast was added to each vial along with three adult females (all day after eclosion) and one male. Flies were incubated at 29°C overnight. The flies were then placed in a new tube, and the total number of eggs was counted.

### RNA isolation

Ovaries were dissected in PBS and homogenized by motorized pestle in 100 μl of TRIzol (Invitrogen, 15596026). RNA was isolated by adding an additional 950 μl of TRIzol and 200 μl of chloroform with mixing. Samples were centrifuged at 13,000 rpm, 4°C for 15 min. The aqueous phase was transferred to a new tube, nucleic acids were precipitated using 1 ml of 100% ethanol and 52 μl of 3 M sodium acetate and precipitated for >1 hour at −20°C. Samples were centrifuged at 13,000 rpm, 4°C for 20 min. Ethanol was decanted, pellets were washed twice with 1 ml of 70% ethanol and dried at room temperature for 10 min. Pellets were dissolved in 20 μl of RNase-free water and placed in a 42°C water bath for 10 min. The concentration of nucleic acid samples was measured on a spectrophotometer. The samples were treated with DNase (TURBO DNA-free Kit, Life Technologies, AM1907) and incubated at 37°C for 30 min. DNase was inactivated using the included DNase. Inactivation reagent and buffer were used according to the manufacturer’s instructions.

### RNA-seq and polysome-seq library preparation

RNA was isolated as previously described above. Total RNA samples were run on a 1% agarose gel to assess sample integrity. To generate RNA-seq libraries, total RNA was incubated with poly(A) selection beads. mRNA libraries were prepared using the NEXTflex Rapid Directional RNAseq Kit (BioO Scientific Corp.). Fragmentation of the mRNA was achieved via incubation at 95°C for 13 min to produce ~300–base pair (bp) fragments. Single-end mRNA sequencing (mRNA-seq) (75 bp) was performed for each sample with an Illumina NextSeq500, carried out by the Center for Functional Genomics (CFG). The sequenced reads were aligned to the *Drosophila melanogaster* genome (UCSCdm6) using HISTAT2 with Refseq annotate transcripts as a guide. featureCounts was used to generate raw counts, and differential gene expression was assayed by DESeq, using a false discovery rate of 0.05, and genes with twofold or greater change in mutant condition compared to control were considered significant. GO enrichment of differential genes was performed using Panther.

Polysome profiling of ovaries was adapted from previous protocols (*44*). Approximately 100 YWT flies (*UAS-Dcr2*;*nosGAL4*) or about 275 experimental ovary pairs of Nop60B were dissected (within 2 hours of eclosion) in PBS. The ovaries were immediately flash-frozen on liquid nitrogen. Samples were homogenized by motorized pestle in lysis buffer, and 20% of lysate was used as input for mRNA isolation and library preparation (as described above). Samples were loaded onto 10 to 45% cycloheximide-supplemented sucrose gradients in 9 of 16 × 3.5 PA tubes (Beckman Coulter, #331372) and spun at 35,000*g* in an SW41 rotor for 3 hours at 4°C. Gradients were fractionated with a density gradient fractionation system. RNA was extracted using acid phenol–chloroform and precipitated overnight. Pelleted RNA was resuspended in 20 μl of water and treated with TURBO DNase, and libraries were prepared as described above.

### Polysome-seq analysis

Analysis of polysome-seq was done using RIVET (*56*). Polysome-associated targets were further defined using the following parameters. Lowly associated mRNAs were identified by ≤2 fold change and *P* value <0.05, while highly associated mRNAs were identified by >2 fold change and *P* value <0.05.

### Ribo-seq library preparation

Ribosome footprinting was performed as previously described (*57*) with several modifications. About five hundred microliters of ovaries was hand-dissected in Schneider’s *Drosophila* Medium (Thermo Fisher Scientific), washed twice in 1 ml of lysis buffer [0.5% Triton X-100, 150 mM NaCl, 5 mM MgCl_2_, and 50 mM tris-HCl (pH 7.5)], and flash-frozen in 2 ml of lysis buffer supplemented by 1 mM dithiothreitol, 50 μM GMP-PNP , emetine (2 μg/ml), and Superase·In RNase Inhibitor (20 U/ml; Ambion) in liquid N_2_. Ovaries were lysed using a Cellcrusher tissue pulverizer (Cellcrusher), allowed to thaw on ice, and centrifuged first at 10,000 rpm for 10 min and then at 13,200 rpm for 10 min. Three hundred microliters of supernatant was used for footprint library preparation, and another 300 μl was used for poly(A)-selected mRNA-seq library preparation. Ribosome footprints were generated by incubating the lysate with 3 U/μg of micrococcal nuclease (New England Biolabs) for 40 min at 25°C and then quenching by the addition of EGTA to a final concentration of 6.25 mM. Ribosomes were sedimented through a 34% sucrose cushion for 2.5 hours at 33,000 rpm in a Beckman SW50 rotor, and the pellet was resuspended in 10 mM tris (pH 7.0). RNA was extracted using TRIzol LS (Invitrogen) and size-selected (28 to 34 nucleotides) on a 15% tris-borate EDTA (TBE)–urea gel. RNA was then dephosphorylated by incubating with T4 polynucleotide kinase (NEB) for 1 hour at 37°C, size-selected, and ligated to the 3′ adapter by incubating with T4 RNA ligase 2 truncated mutant (New England Biolabs) and 1 μg of preadenylated adapter (5′rAppCTGTAGGCACCATCAAT/3ddc) for 2 hours at 25°C. The ligation products were size-selected on a 10% TBE-urea gel. Reverse transcription was performed with Superscript III (Invitrogen) using the Illumina Tru-Seq RT primer:

/5Phos/AGATCGGAAGAGCGTCGTGTAGGGAAAGAGTGTAGATCTCGGTGGTCGC

/iSp18/CACTCA/iSp18/TTCAGACGTGTGCTCTTCCGATCTATTGATGGTGCCTACAG

and the reaction was quenched by incubating with 0.1 M NaOH for 20 min at 98°C. Following rRNA depletion, complementary DNA (cDNA) libraries were circularized by two sequential CircLigase (Epicentre) reactions and amplified by 9 to 12 polymerase chain reaction (PCR) cycles.

### mRNA-seq library preparation

Total RNA was extracted from 300 μl of lysate with TRIzol LS, precipitated with isopropanol, washed in ice-cold 80% ethanol, and resuspended in 10 mM tris-HCl (pH 7.0). mRNA-seq libraries were then prepared from poly(A)-selected mRNA according to the manufacturer’s instructions using the Illumina Tru-Seq RNA Library Prep Kit.

### Processing of Ribo-Seq data

All steps were performed on the Princeton Galaxy server (galaxy.princeton.edu). Multiplexed libraries were demultiplexed using the Barcode Splitter tool with up to two mismatches. Illumina Tru-Seq adapters were clipped using the Trim Galore! tool. The trimmed reads were first mapped against *Drosophila* rRNA sequences using Bowtie with default parameters, and the unaligned reads were then aligned to the *Drosophila* genome Release 6 (dm6) using Bowtie2 with default parameters. The resulting BAM files were used for subsequent analyses.

### Peak detection

The *D. melanogaster* genome (dm6) was divided into 30-bp tiles, and the number of reads aligned to each tile was reported using the bamCoverage tool of the deepTools 2 programming suite. Resulting bedgraph files were preprocessed to break up 30-bp tiles into 30 1-bp tiles (Script1). Peak detection was then performed in R using the Bioconductor software suite. Tiles were first aligned to the transcript regions by gene using the TxDb.Dmelanogaster.UCSC.dm6.ensGene annotation, rtracklayer, GenomicRanges, and BioPhysConnectoR R packages (Script2). Then, the distribution of coverage in the tiles aligned to each gene transcript region was fit to a normal distribution using the MASS R package (Script3 and Function1). Last, the coverage distribution and tiles aligned to each gene region were used to identify peak containing tiles (Script4 and Function2). Peak tiles from different ribosome profiling libraries were then compared (Function3) and the names, locations, and actual sequences of high confidence peaks were extracted (Script5, Script6, and Function4) using the Bsgenome.Dmelanogaster.UCSU.dm6 annotation and the Biostrings and GenomicRanges R packages. Peaks present in at least two of the three Ribo-Seq libraries but not in the control RNA-seq libraries at the corresponding positions were considered high-confidence ribosome footprint peaks.

### Mass spectrometry

Ovaries were dissected in PBS and homogenized by a motorized pestle in 100 μl of TRIzol (Invitrogen, 15596026). RNA was isolated by adding an additional 950 μl of TRIzol and 200 μl of chloroform with mixing. Samples were centrifuged at 13,000 rpm, 4°C for 15 min. The aqueous phase was transferred to a new tube. Nucleic acids were precipitated by adding an equal volume of 5 M ammonium acetate (Sigma-Aldrich) and 2.5 volumes of 100% ethanol and precipitated for >1 hour at −80°C. Samples were centrifuged at 13,000 rpm, 4°C for 20 min. Ethanol was decanted, and pellets were washed four times with 1 ml of cold 70% ethanol and dried at room temperature for 10 min. Pellets were dissolved in 20 μl of RNase-free water and placed in a 42°C water bath for 10 min.

RNA concentration was determined by using UV 260 nm. The RNA was then treated with nuclease P1 and phosphodiesterase to obtain the desired ribonucleotide monophosphate mixtures for mass spectrometric analysis, as previously described (*58*–*60*).

Immediately before analysis, the obtained mononucleotide mixtures were diluted to 4 ng/μl in 10 mM ammonium acetate and 10% isopropanol. All samples were analyzed on a Thermo Scientific LTQ-Orbitrap Velos instrument as previously described (*58*–*60*). Analyses were accomplished using direct infusion electrospray ionization in negative ion mode.

The relative abundance of each RNA PTM was expressed as Abundance versus Proxy (AvP), which was calculated according to the following equation: ${\mathrm{AvP}}_{x}=\frac{{\mathrm{ai}}_{x}}{\sum_{1}^{4}{\mathrm{cr}}_{i}}\times 100$ in which the signal intensity (ai_x_) of each RNA PTM was normalized against the sum of the intensities displayed in the same spectrum by the four canonical bases (cr_i_).

The RNA PTM profiling table translates relative abundances in AvP units to a hot-cold color gradient. The relative abundances displayed by the samples in the first column on the left were used as the baseline for comparisons with the rest of the samples. A different color was assigned only if the respective values were statistically different according to an unpaired Student’s *t* test with a *P* value <0.05. Each data point was the result of three to five biological replicates, which were each separately analyzed three times (technical replicates). Therefore, each value represents the average and SD of a total of 9 to 15 separate analyses.

Tandem mass spectrometry was carried out in negative mode to differentiate uridine and pseudouridine (*58*–*60*). The contribution of each isomer to the initial signal can be estimated from the relative intensities of their unique fragments. The abbreviations and complete names of each PTM in this study are available from the MODOMICS (http://genesilico.pl/modomics/) database.

### Germline ribosome pulldowns

Ribosomal pulldowns were performed as previously described with the following modifications (*40*). Approximately 50 YWT ovaries (*UAS-Dcr2*;*nosGAL4*) and ~100 Nop10^55194^ RNAi ovaries were dissected in PBS. After lysis in ribosomal lysis buffer, 120 μl was collected for input, and TRIzol extraction was performed as previously described for mass spectrometry. The remaining lysate was divided into 180-μl aliquots. Six micrograms of rabbit immunoglobulin G (IgG) (Jackson ImmunoResearch) or rat-HA antibodies were added to the lysate for 3 hours with rotation at 4°C. At hour 2, 50 μl of Dynabeads A (Thermo Fisher Scientific) was added per replicate. The beads were prepped by performing four washes using a magnetic rack (500 μl for 2 min each) with ribosomal lysis buffer. After the fourth wash, the beads were resuspended in 50 μl of ribosomal lysis buffer. To the samples, either 25 μl of IgG or anti-HA was added and left overnight with rotation at 4°C. The following day, the beads were washed with 200 μl of ribosome lysis buffer for a total of four washes. After the final wash, the beads were resuspended in 15 μl of ribosome lysis buffer. A TRIzol extraction was performed as previously described for mass spectrometry. After RNA extraction, a small portion of the RNA was run on a 1% agarose gel to confirm the presence of rRNA.

After the overnight incubation, the beads were washed with 200 μl of ribosome lysis buffer for a total of four washes. After the final wash, the beads were resuspended in 15 μl of ribosome lysis buffer. To the sample, 4× SDS buffer was added and then heated at 95°C for 5 min and stored at −20°C until Western blot analysis.

### Western blot

Ovaries were dissected in PBS (*44*). After dissection, PBS was aspirated, and 30 μl of radioimmunoprecipitation assay (RIPA) buffer with protease inhibitors was added, and the tissue was homogenized. The homogenate was centrifuged at 13,000 rpm for 15 min at 4°C. The aqueous layer was transferred into a new tube while avoiding the top lipid layer. One microliter of the protein extract was used to determine protein concentration via the Invitrogen Qubit Protein Assay Kit. Protein (15 to 20 μg) was denatured with 4× Laemmli sample buffer and β-mercaptoethanol at 95°C for 5 min. The samples were loaded onto a Mini-PROTEAN TGX 4 to 20% gradient SDS–polyacrylamide gel electrophoresis gels and run at 300 V for 20 min. The proteins were then transferred to a 0.20-μm nitrocellulose membrane using the Bio-Rad Trans-blot Turbo System. After the transfer, the membrane was blocked in 5% nonfat dry milk in PBST for 2 hours at room temperature. The following antibodies were used: rat-HA (1:4000), rabbit-Vasa (1:6000), and rabbit-RpL26 (1:1000). Primary antibody was prepared in 5% milk in PBST and was added to the membrane and incubated at 4°C overnight. The membrane was then washed three times in 0.5% milk in PBST. Anti-rabbit horseradish peroxidase (HRP) (1:10,000) or anti-rat HRP (1:10,000) was prepared in 5% milk in PBST and was added to the membrane and incubated at room temperature for 1 hour. The membrane was then washed three times in PBST. The Bio-Rad chemiluminescence ECL kit was used to image the membrane.

Note that to help normalize the germline in the Western blot probing for the polyQ-HA reporter, the first 15 to 20 μg of lysate was run and probed for Vasa. Controls were then diluted 1:5 to help equalize the amount of germline loaded and compared to the H/ACA snRNP complex member knockdown. Normalizations were performed using the top Vasa band.

### Pseudouridine pulldowns

Ovaries were dissected in PBS (*44*). After dissection, PBS was aspirated and 100 μl of RIPA buffer containing 1 μl of RNaseOUT Recombinant Ribonuclease Inhibitor (Invitrogen) was added. The tissue was homogenized and centrifuged for 20 min at 13,000 rpm. From the homogenate, 20% was taken for an input for library prep as described above. The remaining sample was divided into two equal aliquots. To one of the samples, 5 μg of rabbit IgG (Jackson ImmunoResearch) or 5 μg of pseudouridine antibody (see antibody list) was added and left for 16 hours overnight at 4°C with constant rocking. The beads were then washed with 1:10 diluted RIPA buffer + RNaseOUT four times. After the final wash, the beads were resuspended with 20 μl of RIPA buffer + RNaseOUT, and RNA was isolated, and mRNA-seq libraries were created as described above for the input, IgG, and pseudouridine pulldown samples.

### Quantitative real-time polymerase chain reaction

Once RNA was purified and isolated (see the “RNA isolation” section), a reverse transcription was performed using Superscript II according to the manufacturer’s protocol with equivalent volumes of RNA for each sample. cDNA was amplified using 5 μl of SYBR Green Master Mix and 0.3 μl of 10 μM of each reverse and forward primers in a 10-μl reaction. For each sample, three biological and a minimum of two technical replicates were performed. Technical replicates were averaged, and tubulin was used as a control. To calculate fold change relative to tubulin mRNA levels, the average of the 2^−ΔCt^ for the biological replicates was calculated with error bars representing SEM. Statistics were performed using a paired *t* test on ΔCt (Cycle threshold) values. Primers used can be found in primerlist.xslx.

### MEME analyses

The 5′UTR, CDS, 3′UTR, and amino acid sequence of 465 mRNAs that are lowly associated with polysome, and 320 mRNAs highly associated with polysome, compared to control, were analyzed by the MEME algorithm. Discriminative mode analysis was conducted against 1573 nontarget gene sequences as background with default parameters. Motif logos, number of sites, and E-values all reported as produced by the output of the program.

### FIMO analyses

An amino acid motif of 5Qs was run against the amino acid sequences of all mRNAs that were lowly associated with polysomes. Motifs identified in targets were searched in the given strand with a *P* value <1 × 10^−4^.

Percentage of mRNAs with 5× CAG repeats: RNA-seq data were obtained from previous studies conducted by the Rangan laboratory. Data are available via the following GEO accession numbers: >*bam* RNAi;*hs*-*bam* GSE143728. TPMs were averaged for the libraries, and enriched genes that were in the cyst stages were identified as having an average of 1 TPM or greater. A motif of 5 CAGs was run against the CDS sequences of all mRNAs identified as enriched in the cyst stages as previously stated. All targets in the output file were reported in the Excel file and then filtered for the unique number of genes that contained the motif with a *P* value <1 × 10^−4^.

## Supplementary Materials

### Other Supplementary Material for this manuscript includes the following:

Tables S1 to S7

Primers list
