# Supplementary material for: H/ACA snRNP–dependent ribosome biogenesis regulates translation of polyglutamine proteins
Source: Sci Adv. 2023 Jun 21;9(25):eade5492. doi: 10.1126/sciadv.ade5492 (PMC10284551; doi:10.1126/sciadv.ade5492)
Supplement: Supplementary file 1 — Figs. S1 to S13 Legends for tables S1 to S7 [file sciadv.ade5492_sm.pdf]

Science Advances
AAAS

Supplementary Materials for

H/ACA snRNP–dependent ribosome biogenesis regulates translation of polyglutamine proteins

Shane M. Breznak *et al.*

Corresponding author: Prashanth Rangan, prashanth.rangan@mssm.edu; Elizabeth R. Gavis, gavis@princeton.edu

Sci. Adv. **9**, eade5492 (2023)
DOI: 10.1126/sciadv.ade5492

The PDF file includes:

Figs. S1 to S13
Legends for tables S1 to S7

Other Supplementary Material for this manuscript includes the following:

Tables S1 to S7
Primers list

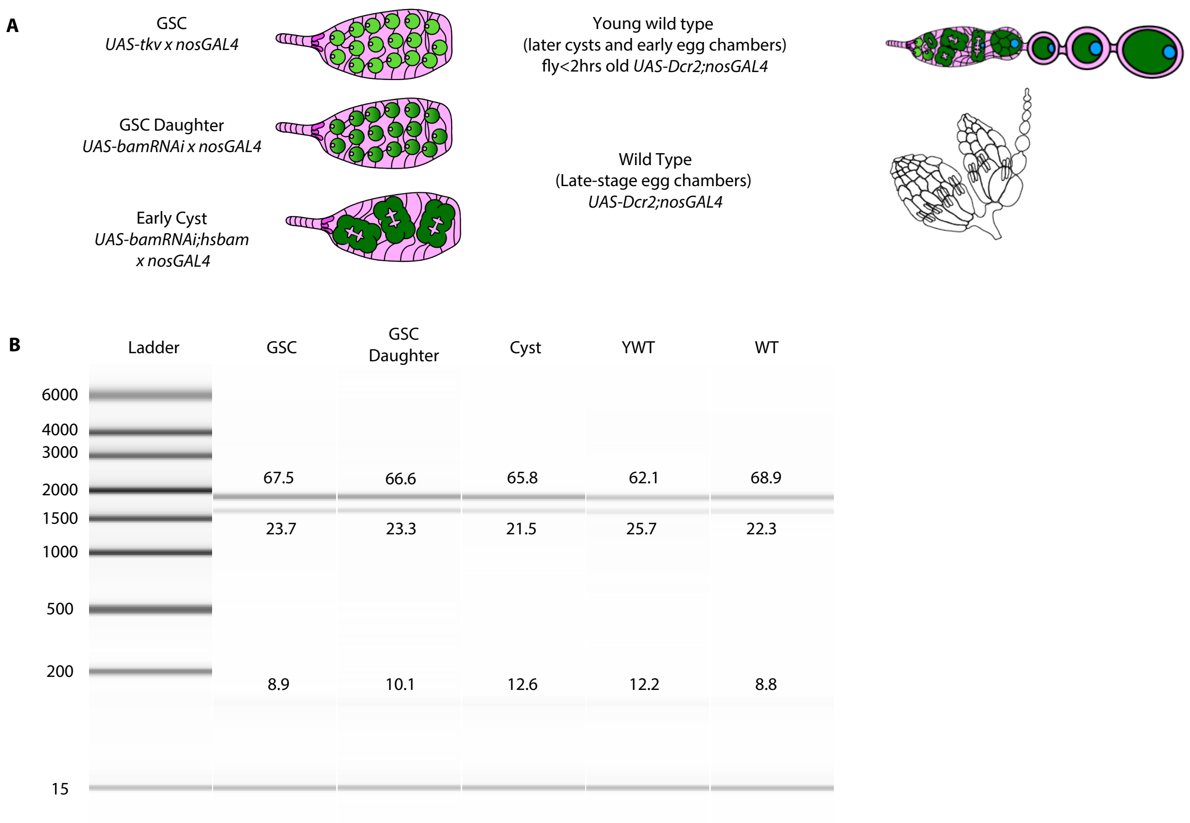

Fig. S1. Relative RNA contributions throughout developmental stages

(A) Method to enrich for developmental stages.

(B) Fragment analyzer of total RNA from different developmental stages showing corresponding RNA contributions to total RNA with 3 bands (18s RNA ~1866, 16s RNA ~1600 and small RNAs ~148). To each lane 40 ng of total RNA was loaded for the indicated developmental stages: GSC, GSC daughter, cyst, YWT and WT. The ladder band sizes are represented by the numbers to the far left. Numbers near bands are representative of relative concentration of the corresponding band, represented as a percentage of the total concentration.

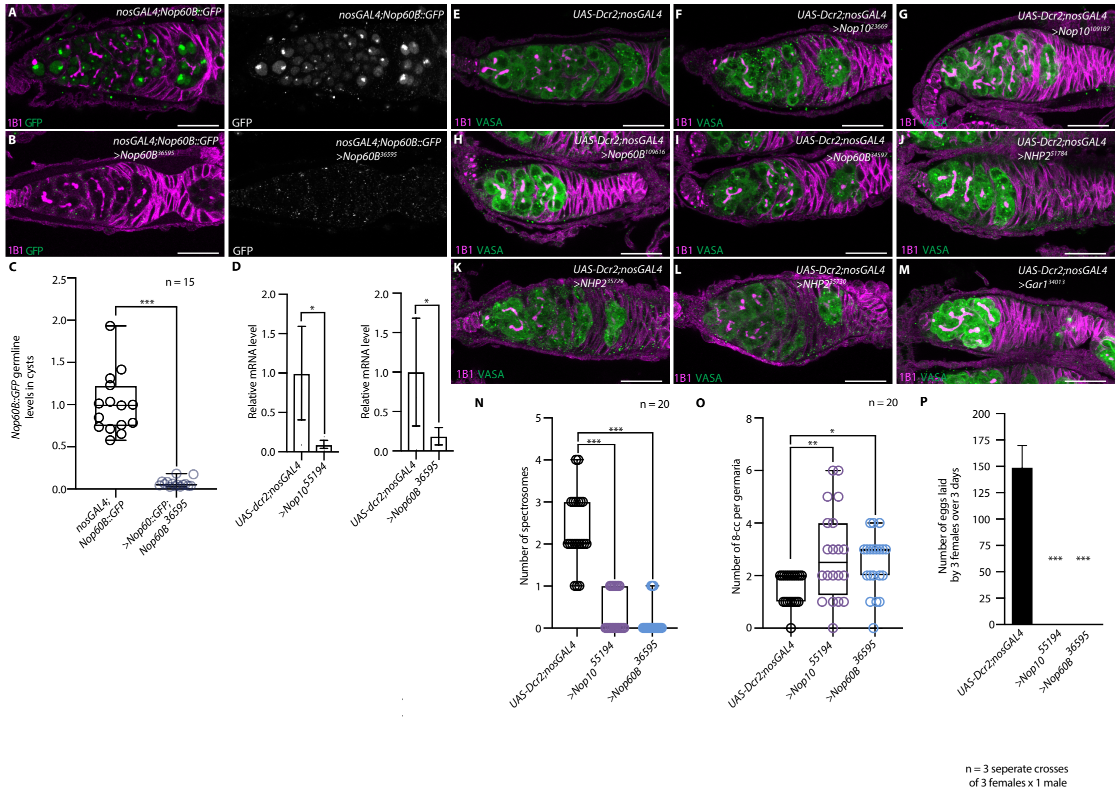

Fig. S2. The H/ACA snRNP complex is required in the germline for proper oogenesis

(A) *nosGAL4;Nop60B::GFP* and *Nop60B* RNAi in *Nop60B::GFP* ovaries (B) stained with anti-1B1 (magenta) and anti-GFP (green/gray). Scale bar is 20 µm.

(C) Unpaired t-test of GFP levels in *nosGAL4;Nop60B::GFP* and germline knockdown of *Nop60B* in *Nop60B::GFP* background (n = 15, *** p<0.0001).

(D) qRT-PCR of *Nop10* or *Nop60B* normalized to *UAS-Dcr2;nosGAL4* (n = 3, Nop10: * p = 0.0231, paired t-test) (n=3, Nop60B: * p = 0.0142, paired t-test, Error bars = SEM).

(E-M) *UAS-Dcr2;nosGAL4* (E) and depletion of H/ACA snRNP complex members (F-M) stained with anti-1B1 (magenta) and anti-Vasa (green). Scale bar is 20 µm.

(N) Number of spectrosomes in *UAS-Dcr2;nosGAL4, Nop10* or *Nop60B* germaria. Dunnett's multiple comparisons test post-hoc test after one-way ANOVA (n = 20, *** p<0.0001).

(O) Number of 8-cell cysts in *UAS-Dcr2;nosGAL4*, *Nop10* and *Nop60B* ovaries. Dunnett's multiple comparisons test post-hoc test after one-way ANOVA ($n = 20$, * $p=0.0341$, ** $p=0.0030$).

(P) Egg laying assay with Dunnett's multiple comparisons post-hoc test after one-way ANOVA. ($n = 0\text{-}173$, *** $p<0.001$, Error bars = SD).

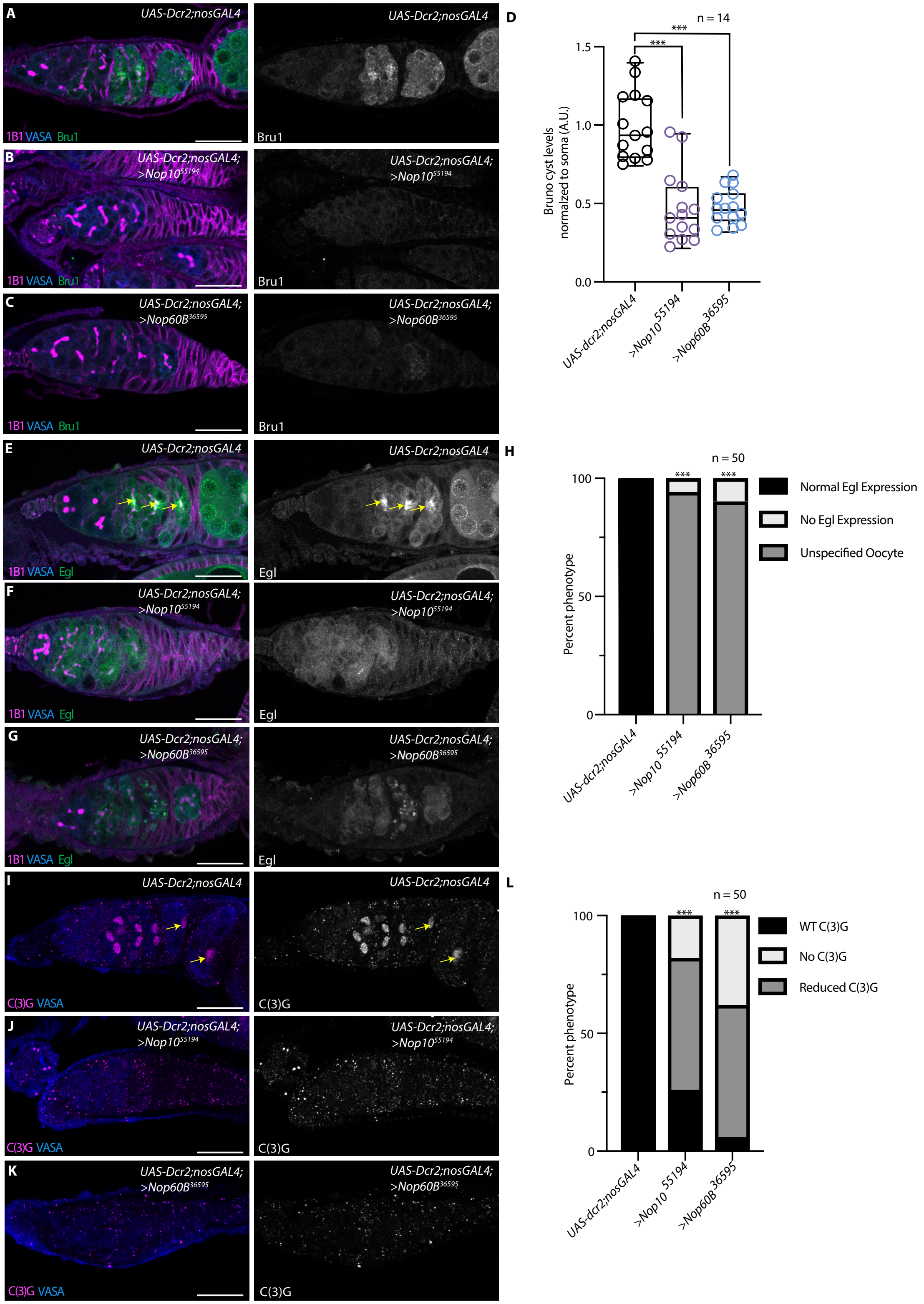

Fig. S3. The H/ACA snRNP complex is required for meiotic progression

(A-C) Germaria of *UAS-Dcr2;nosGAL4* (A), *Nop10* (B) and *Nop60B* (C) ovaries stained with anti-1B1 (magenta), anti-Vasa (blue) and anti-Bru1 (green/gray).

(D) Bru1 levels in *UAS-Dcr2;nosGAL4*, *Nop10* and *Nop60B* germaria normalized to soma. Statistics performed were Dunnett's multiple comparisons test post-hoc test after one-way ANOVA (n = 50 each, *** $p<0.0001$).

(E-G) Germaria of *UAS-Dcr2;nosGAL4* (E), *Nop10* (F) and *Nop60B* (G) ovaries stained with anti-1B1 (magenta), anti-Vasa (blue) and anti-Egl (green/gray). Arrow pointing at designated oocyte. Scale bar is 20 µm.

(H) Quantification of oogenesis defect phenotypes showing a loss of oocyte specification in germaria depleted of the H/ACA box complex. Statistical analysis performed with Fisher's exact test (n = 50 each, *** $p<0.0001$).

(I-K) Germaria of *UAS-Dcr2;nosGAL4* (I), *Nop10* (J) or *Nop60B* (K) ovaries stained with anti-c(3)G (magenta/gray) and anti-Vasa (blue). Arrow points to designated oocyte. Scale bar is 20 µm.

(L) Quantification of oogenesis defects in germaria depleted of the H/ACA box complex. Statistical analysis performed with Fisher's exact test (n = 50 each, *** $p<0.0001$).

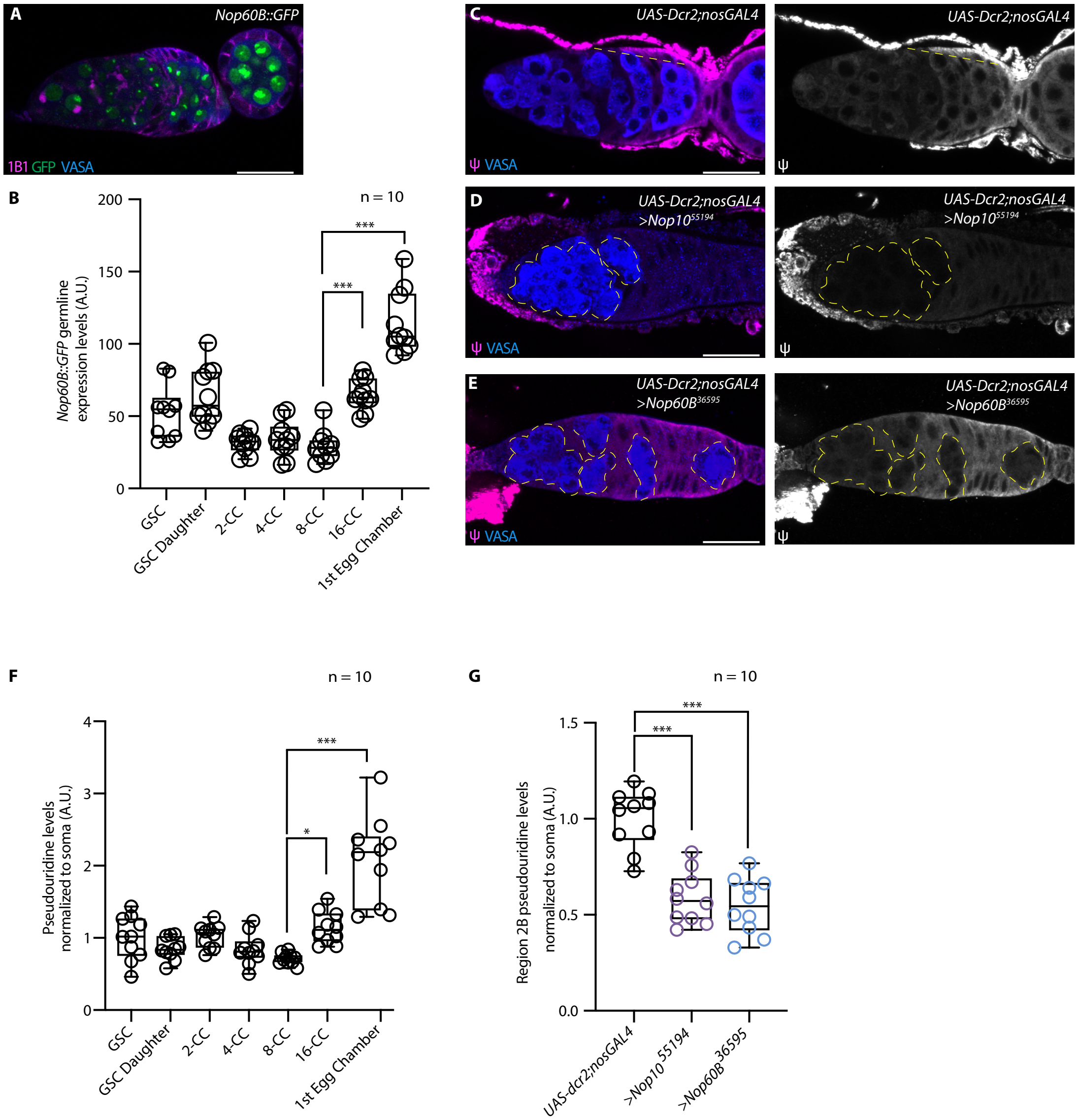

Fig. S4. Nop60B and pseudouridine increase during transition from cyst to egg chamber

(A) *Nop60B::GFP* germarium stained with anti-1B1 (magenta), anti-Vasa (blue) and anti-GFP (green/gray). Scale bar is 20 µm.

(B) Quantitation of GFP from GSC to 1st egg chamber in *Nop60B::GFP* ovary. Statistics performed were Tukey's multiple comparisons post-hoc test after one-way ANOVA. Statistics shown comparing 8-cell cyst to 16-cell cyst and to the egg chamber (n = 10, *** p<0.001).

(C-E) Germaria of *UAS-Dcr2;nosGAL4* (C), *Nop10* (D) and *Nop60B* ovaries (E) stained with anti-pseudouridine (magenta/gray) and anti-Vasa (blue). Yellow dotted line in control represents area of increasing pseudouridine levels while yellow outline in *Nop10* and *Nop60B* represents loss of pseudouridine. Scale bar is 20 µm.

(F) Pseudouridine levels from GSC to 1st egg chamber in *UAS-Dcr2;nosGAL4* ovary. Statistics performed were Tukey's multiple comparisons post-hoc test after one-way ANOVA. Statistics shown comparing 8-cell cyst to 16-cell cyst and to the egg chamber (n = 10, * $P < 0.0313$, *** $p < 0.001$).

(G) Pseudouridine levels in 2B-region of *UAS-Dcr2;nosGAL4* and *Nop10* or *Nop60* ovaries normalized to soma (n = 10, *** $p < 0.001$).

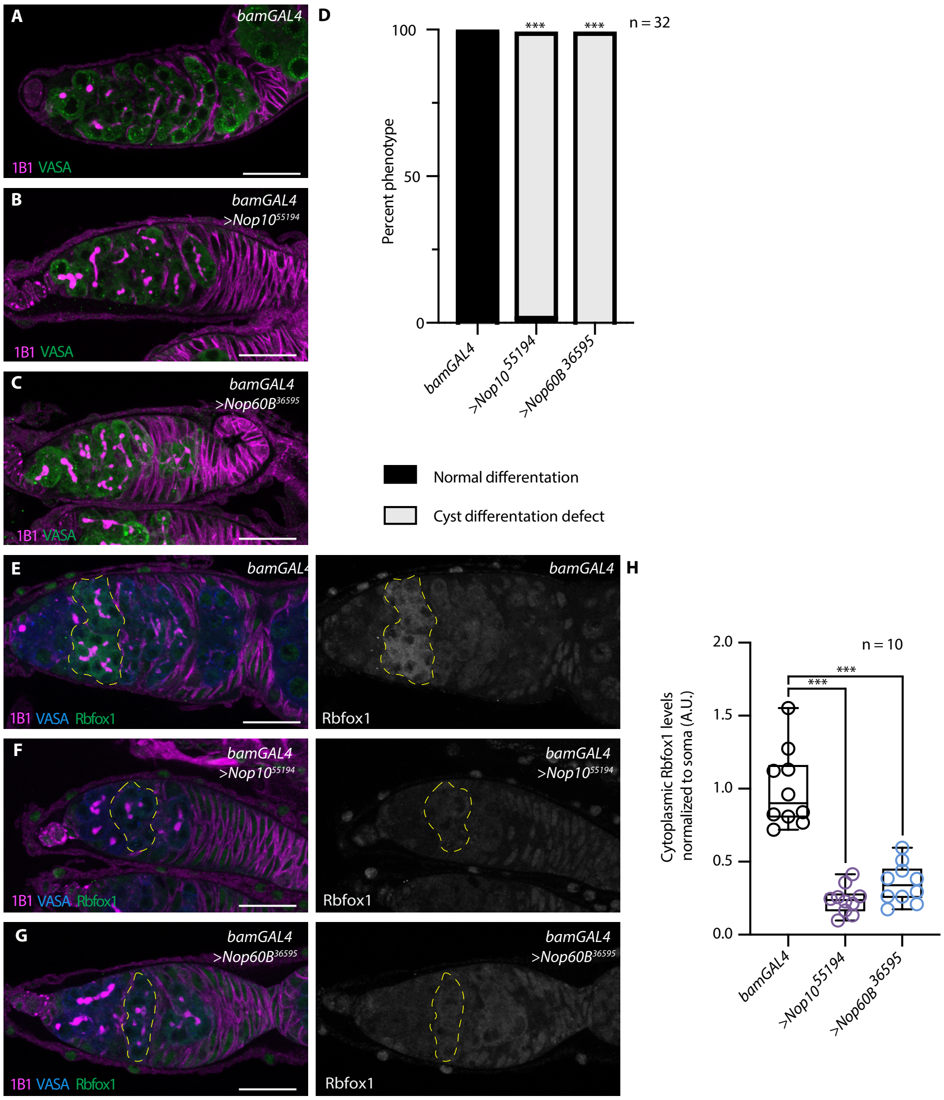

Fig. S5. The H/ACA snRNP complex is required in the cyst stages

(A-C) Germaria of *bamGAL4* (A), *Nop10* (B) or *Nop60B* ovaries (C) stained with anti-1B1 (magenta) and anti-Vasa (green). Scale bar is 20 µm.

(D) Quantification of oogenesis defect phenotypes per genotype. Statistical analysis performed with Fisher's exact test (n = 32 for all, *** p<0.0001).

(E-G) Germaria of *bamGAL4*, *Nop10* (F) and *Nop60B* ovaries (G) stained with anti-1B1 (magenta), anti-Vasa (blue) and anti-Rbfox1 (green/gray). Scale bar is 20 μm for all images. Yellow dotted line outlined cysts.

(H) Quantification of Rbfox1 levels in *Nop10* and *Nop60B* depleted germaria. Statistical analysis performed with Dunnett's multiple comparisons post-hoc test after one-way ANOVA, ($n = 10$ each, *** $p<0.0001$).

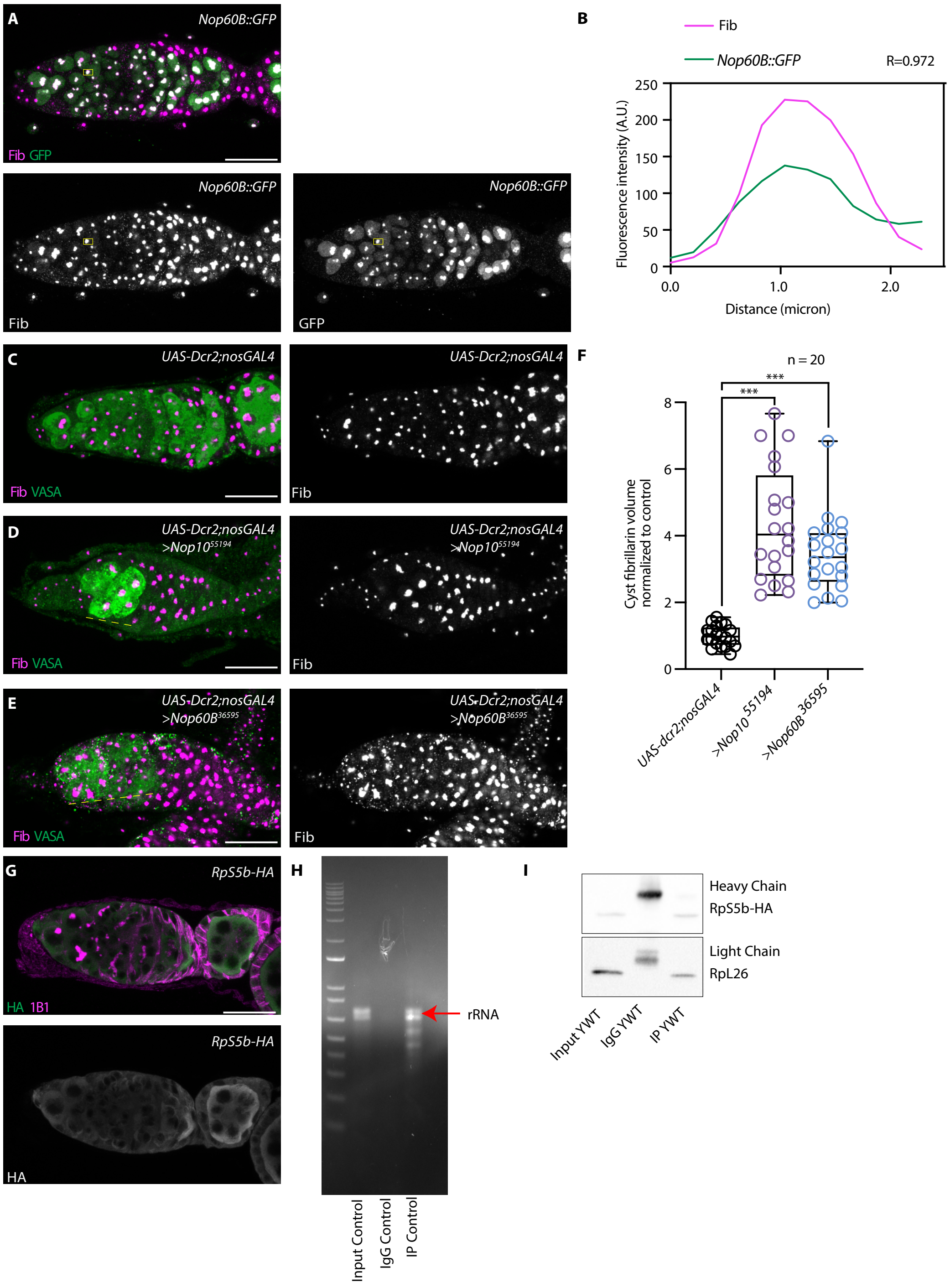

Fig. S6. The H/ACA snRNP complex deposits pseuoduiridne on rRNA and is required for proper ribosome biogenesis

(A) *Nop60B::GFP* germarium stained with Fibrillarin (magenta/gray) and GFP (green/gray). Scale bar is 20 µm.

(B) Fluorescence intensity plot generated from a box of averaged pixels centered around the punctate of Fibrillarin in the yellow box. R values denote Spearman correlation coefficients between GFP and Fibrillarin.

(C-E) Germaria of *UAS-Dcr2;nosGAL4*, *Nop10* (D) and *Nop60B* (E) ovaries stained with fibrillarin (magenta/gray) and Vasa (green). Scale bar is 20 µm.

(F) Quantification of nucleolar volume in the cysts stages per genotype. Statistics performed were Dunnett's multiple comparisons post-hoc test after one-way ANOVA ($n = 20$ each, *** $p<0.0001$).

(G) Germarium of *RpS5b-HA* ovary stained with anti-1B1 (magenta) and anti-HA (green/gray).

(H) Agarose gel of control lysate showing pulldown enrichment of rRNA (red arrow).

(I) Western blot analysis of ribosomal pulldowns probing for HA and RpL26 in input, IgG and pulldown samples.

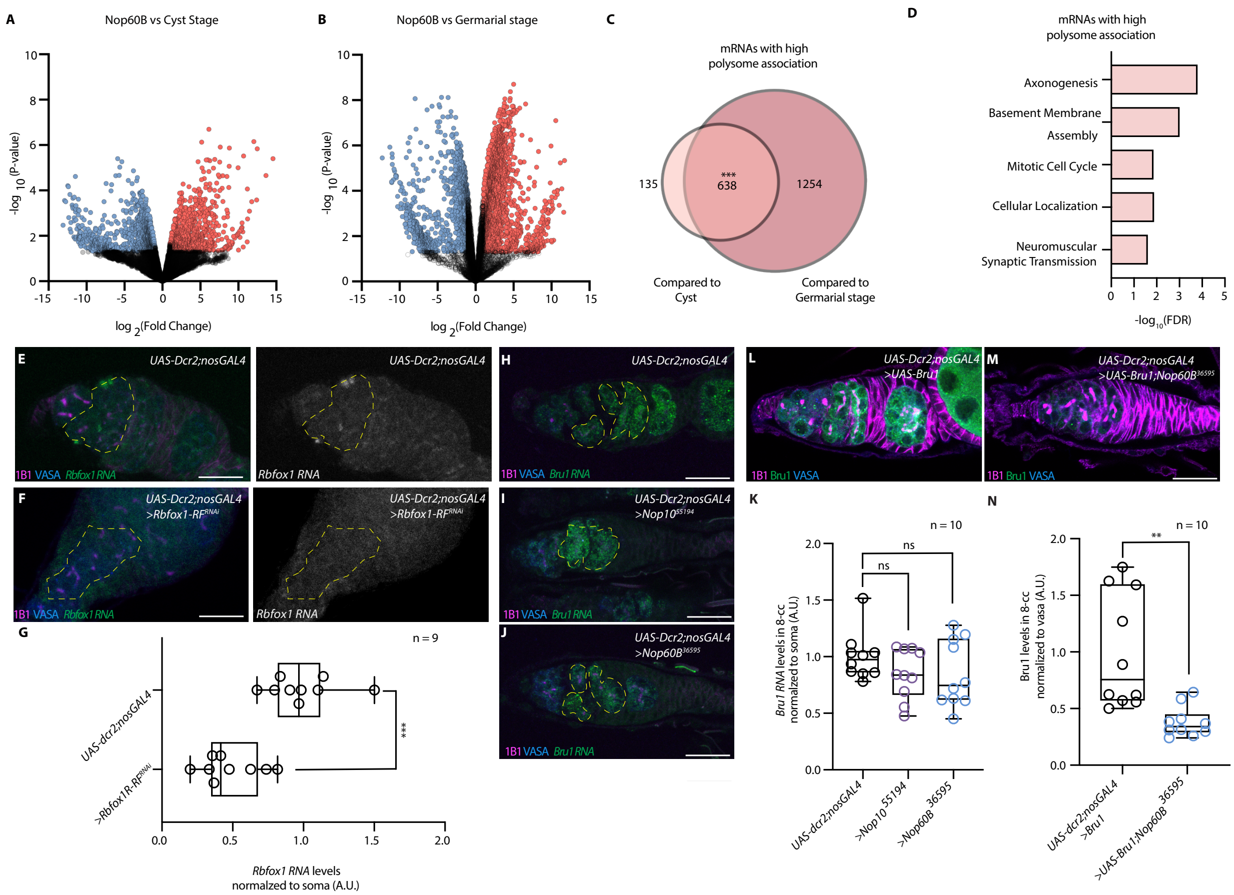

Fig. S7. The H/ACA snRNP complex is required for translation of meiotic mRNAs

(A-B) Volcano plot of *Nop60B* vs cyst stages (A) and *Nop60B* vs germarial stages (YWT) (B) showing mRNAs that have low and high association with ribosomes (n = 2, targets identified as 2-fold cutoff).

(C) Venn diagram illustrating overlapping targets of Nop60B-polysome >2 fold (significance to low to compute using RStudio using Hypergeometric Test). Germarial stages consist of ovaries of young *UAS-Dcr2;nosGAL4* while the cyst stage consist of *bamRNAi;hsbam* which enrich for cyst stages in development (enrichment described in methods).

(D) Significant biological process GO terms of shared highly associated mRNAs in *Nop60B*.

(E) In situ hybridization to *Rbfox1* RNA (green/gray), anti-1B1 (magenta) and anti-Vasa (blue) in *UAS-Dcr2;nosGAL4* (E) and *Rbfox1-RF*^{RNAi} (F) ovaries. Scale bar is 20 µm. Yellow dotted line outlines *Rbfox1* RNA.

(G) Quantification of *Rbfox1* levels in *UAS-Dcr2;nosGAL4* and *Rbfox1-RF*^{RNAi} ovaries normalized to soma. Statistics performed were unpaired t-test (n = 9 each, ***, p=0.0002).

(H-J) In situ hybridization to *bru1* RNA (green/gray), anti-1B1 (magenta) and anti-Vasa (blue) in *UAS-Dcr2;nosGAL4* (H), *Nop10* (I) and *Nop60B* (J). Scale bar is 20 µm. Yellow dotted line outlines *bru1* RNA.

(K) Quantification of *bru1* RNA levels in *Nop10* and *Nop60B* ovaries normalized to soma. Statistics performed were Dunnett's multiple comparisons post-hoc test after one-way ANOVA (n = 10 each, ns, p=0.3606 and p=0.3752 respectively).

(L-M) The expression of the transgene, *UAS-bru1*, driven in the germarium of *UAS-Dcr2;nosGAL4* (I) and (J) *Nop60B* ovaries (J). Scale bar is 20 µm.

(N) Quantification of *bru1* levels normalized to Vasa. Statistics performed were unpaired t-test (n = 10 each, ** p=.0015).

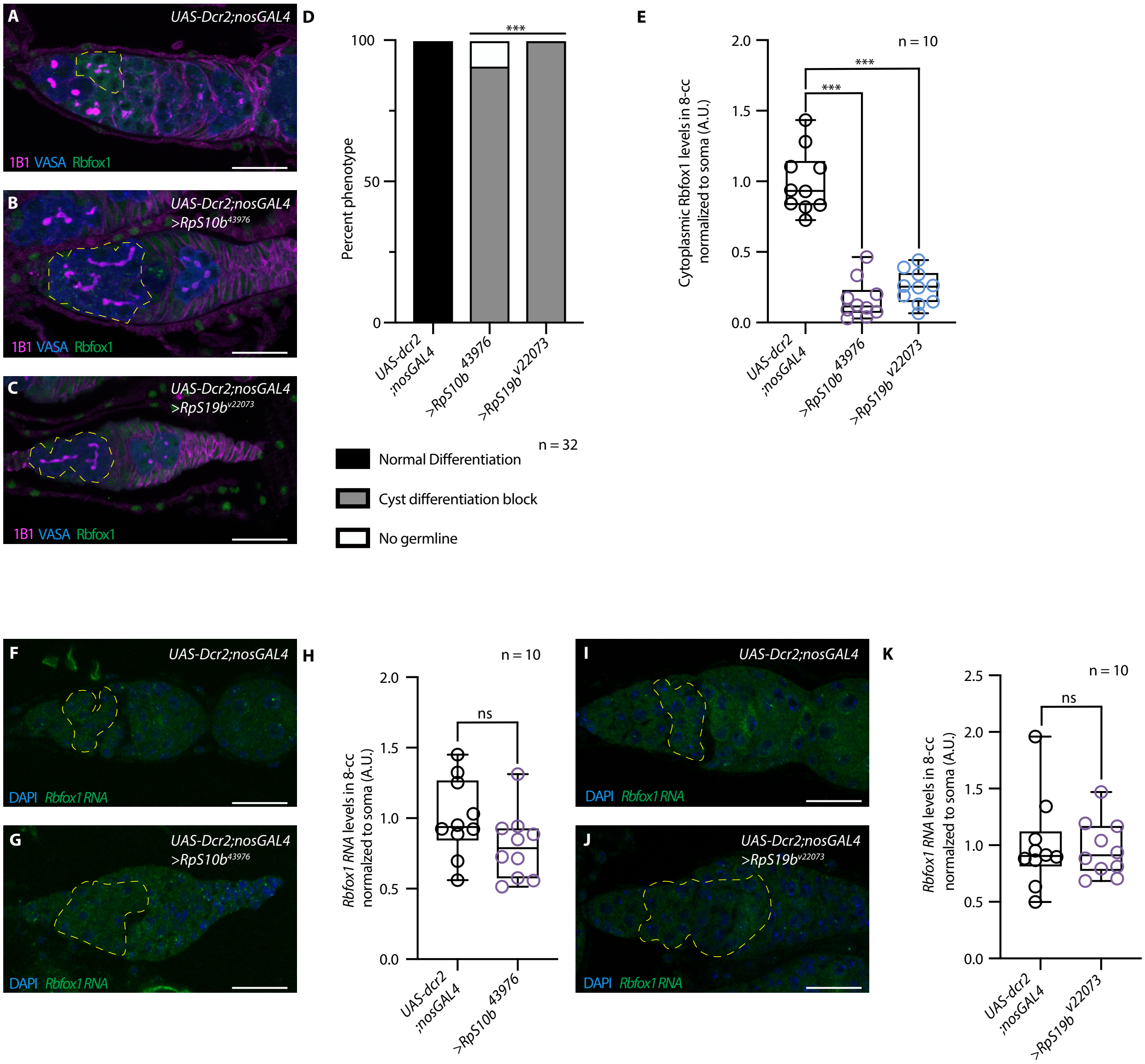

Fig. S8. Ribosomal paralogs are required for Rbfox1 translation

(A-C) *UAS-Dcr2;nosGAL4* (A), *RpS10b* (B) and *RpS19b* (C) ovaries stained with anti-1B1 (magenta), anti-Vasa (blue) and anti-Rbfox1 (green). Scale bar is 20 µm. Yellow dotted lines outline cysts.

(D) Quantification of oogenesis defect phenotypes per genotype. Statistical analysis performed with Fisher's exact test (n = 32 for all, *** p<0.0001).

(E) Quantification of cytoplasmic Rbfox1 levels normalized to soma in germline depletion of *RpS10b* and *RpS19b*. Statistics performed were Dunnett's multiple comparisons post-hoc test after one-way ANOVA (n = 10 each, *** p<0.0001).

(F-G) In situ hybridization of *Rbfox1* RNA (green) and DAPI (blue) in *UAS-Dcr2;nosGAL4* (F) and *RpS10b* (G) ovaries. Scale bar is 20 µm. Yellow dotted line outlines *Rbfox1* RNA.

(H) Quantification of *Rbfox1* RNA levels in *RpS10b* ovaries normalized to soma. Statistics performed were unpaired t-test (n = 10 each, ns, p=0.1006).

(I-J) In situ hybridization of *Rbfox1* RNA (green) and DAPI staining (blue) in *UAS-Dcr2;nosGAL4* (I) and *RpS19b* (J) ovaries. Scale bar for all images is 20 µm. Yellow dotted line outlines *Rbfox1* RNA.

(K) Quantification of *Rbfox1* RNA levels in *RpS19b* ovaries normalized to soma. Statistics performed were unpaired t-test (n = 10 each, ns, p=0.8258).

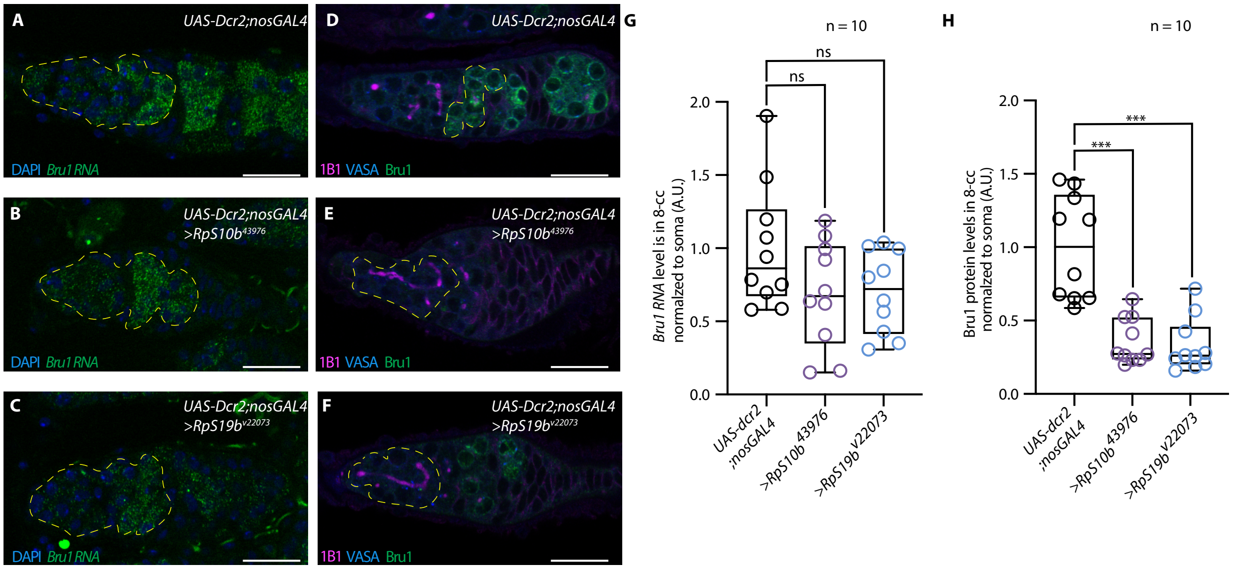

Fig. S9. Ribosomal paralogs are required for *bru1* translation

(A-C) In situ hybridization to *bru1* RNA (green) and DAPI staining (blue) in *UAS-Dcr2;nosGAL4* (A), *RpS10b* (B) and *RpS19b* (C) ovaries. Scale bar is 20 µm. Yellow dotted line outlines *bru1* RNA.

(D-F) Germarium of *UAS-Dcr2;nosGAL4* (D), *RpS10b* (E) and *RpS19b* (F) stained with anti-1B1 (magenta), anti-Vasa (blue) and anti-Bru1 (green). Scale bar is 20 µm. Yellow dotted line outlines cysts.

(G) Quantification of *bru1* RNA levels normalized to soma in *RpS10b* and *RpS19b* ovaries. Statistics performed were Dunnett's multiple comparisons post-hoc test after one-way ANOVA (n = 10 each, not significant, p=0.1149 and 0.1325, respectively).

(H) Quantification, selected from the region of interest (marked by yellow dotted line), of Bru1 protein levels normalized to soma in *RpS10b* and *RpS19b*. Statistics performed were Dunnett's multiple comparisons post-hoc test after one-way ANOVA (n = 10 each, *** p<0.0001).

A

Flybase symbol	Pseudouridine Synthase	Location
CG7849	TruB/TruB2	Unknown tRNA or mRNA
CG3709	Pus10	tRNA Uracil 55

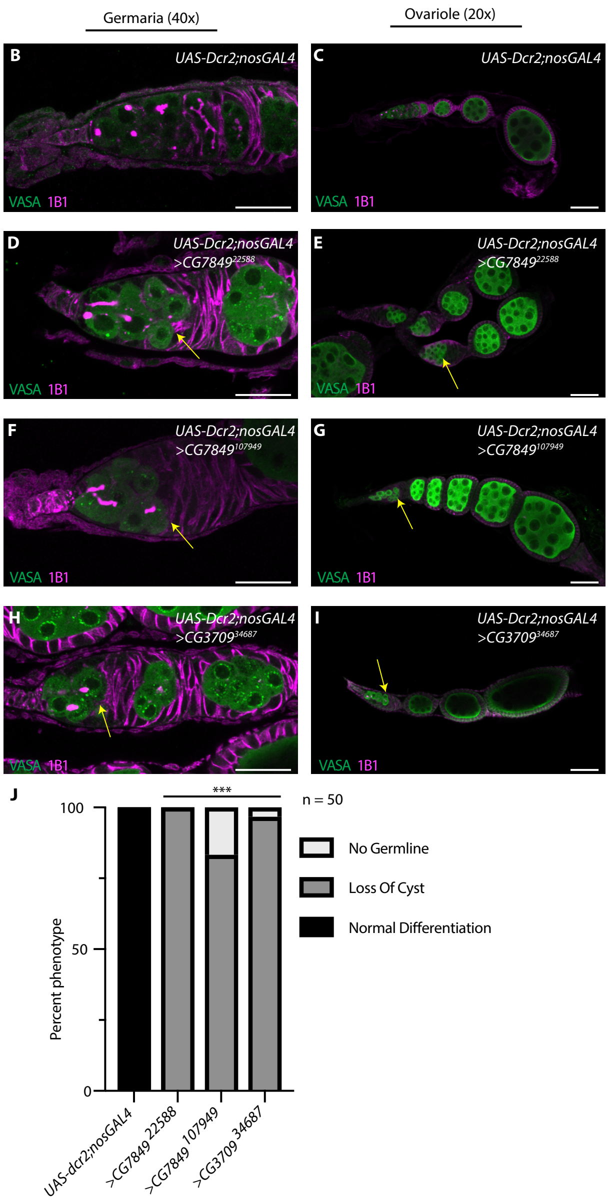

Fig. S10. tRNA pseudouridine synthases are required for differentiation but do not phenocopy loss of rRNA pseudouridine synthases

(A) Table of tRNA pseudouridine synthases and location of pseudouridine deposition found to have a differentiation defect.

(B, C) Images of a germarium at 40x (B) and an ovariole at 20x (C) of *UAS-Dcr2;nosGAL4* ovaries stained with anti-1B1 (magenta) and anti-Vasa (green).

(D, E) Images at 40x (D) and 20x (E) of germarium where *CG7849* is depleted in the germline and stained with anti-1B1 (magenta) and anti-Vasa (green).

(F, G) Images at 40x (F) and 20x (G) of germarium using a second RNAi line to deplete *CG7849* in the germline and stained with anti-1B1 (magenta) and anti-Vasa (green).

(H, I) Images at 40x (H) and 20x (I) of germarium where *CG3709* is depleted in the germline and stained with anti-1B1 (magenta) and anti-Vasa (green). Yellow arrow points to region where cysts are lost in all 20x images. Scale bar is 20 μm.

(J) Quantification of oogenesis defect phenotypes in germline knockdown of tRNA pseudouridine synthases. Statistical analysis performed with Fisher's exact test (n = 50 each, *** $p<0.0001$).

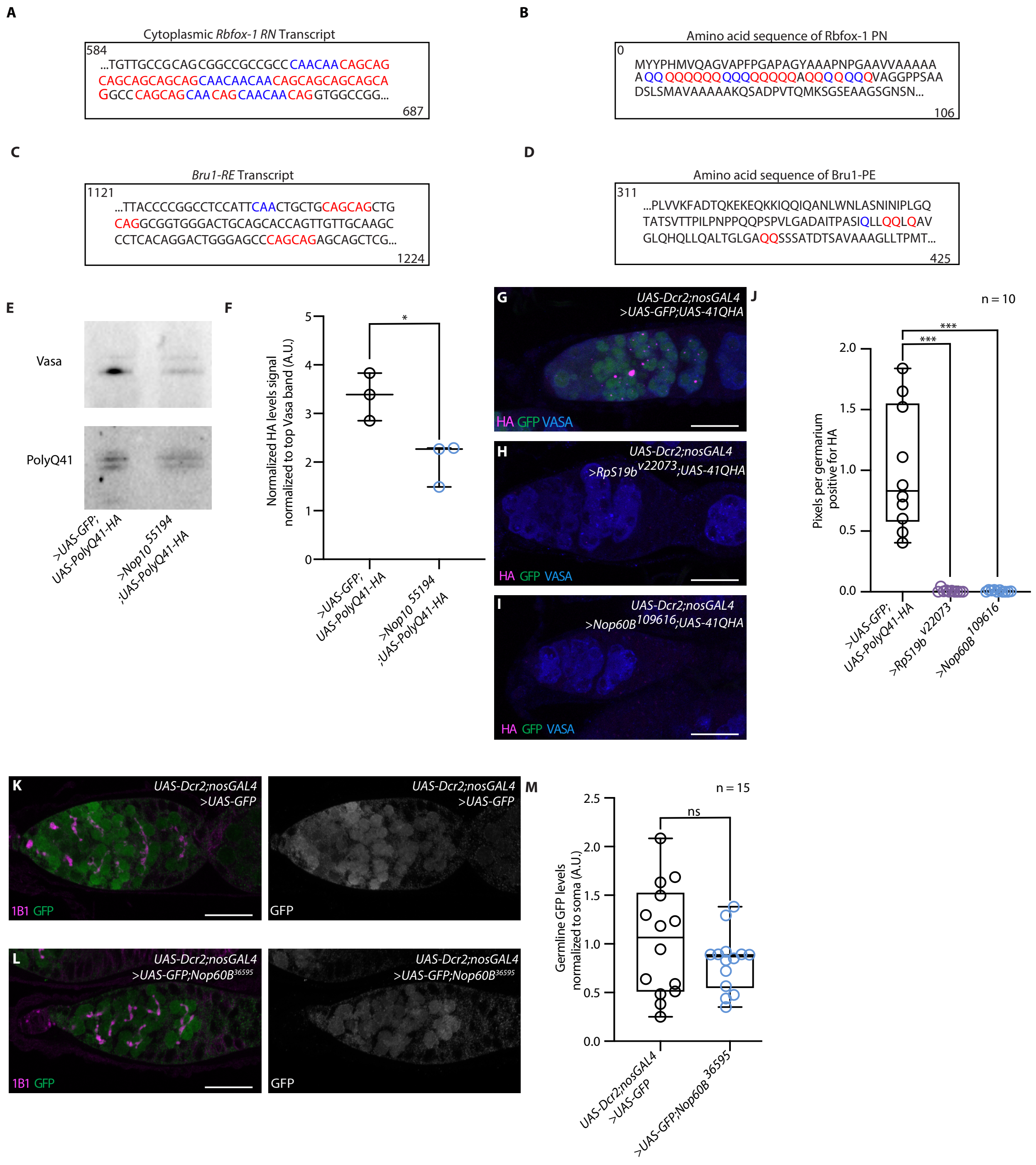

Fig. S11. The H/ACA snRNP complex is required for translating polyQ proteins

(A) *Rbfox1-RN* sequence with glutamine (Q) codons CAA (blue) and CAG (red) and corresponding protein sequence Rbfox1-PN (B).

(C) *bru1-RE* sequence with glutamine (Q) codons CAA (red) and CAG (blue) and corresponding protein sequence Bru1-PE (D).

(E) Western blot of poly41Q-HA in control and *Nop10* ovaries. Western was probed with HA to detect polyQ.

(F) Signal-ratio between the HA and upper Vasa band. Statistics performed were unpaired t-test (n = 3, * p=.0253).

(G-I) Poly41Q-HA reporter in *UAS-Dcr2;nosGAL4, RpS19b* (H) and *Nop60B* (I) ovaries stained with anti-HA (magenta), anti-GFP (green) and anti-Vasa (blue). Scale bar is 20 µm.

(J) HA reporter quantitation using unpaired t-test (n = 10, *** p=0.0001).

(K, L) *UAS-GFP* in control (K) and *Nop60B* (L) ovaries, stained with anti-1B1 (magenta) and anti-GFP (green/gray). Scale bar is 20 µm.

(M) Quantitation of GFP levels normalized to soma. Statistics performed were unpaired t-test (n = 15, ns, p=0.2187).

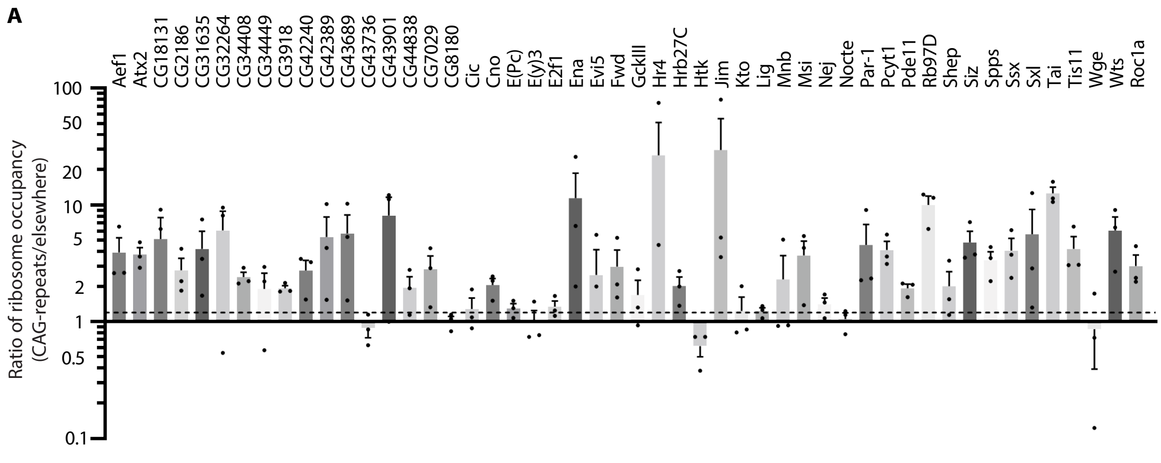

Fig S12. Enrichment of ribosomes at CAG-repeats.

(A) Ribosome occupancy within and outside of CAG-repeats was calculated for all poly-Q (plus additional poly-S and poly-A) tract-containing transcripts identified in the peak-finding analysis.

The ratio of ribosome occupancy on the region containing CAG-repeats ± 50-nt flanking sequences to ribosome occupancy on the remainder of the transcript was plotted for each transcript with ribosome occupancy in at least two of the three Ribo-Seq experiments. 45 out of 50 transcripts showed over 20% increase (dotted line) in ribosome occupancy at CAG-repeats; 34 out of 50 transcripts showed over 2-fold increase. Ribosome occupancy was defined as: # of Ribo-Seq tags / # of RNA-Seq tags. Values are mean + SEM for means > 1 or mean – SEM for means < 1 from three Ribo-Seq experiments.

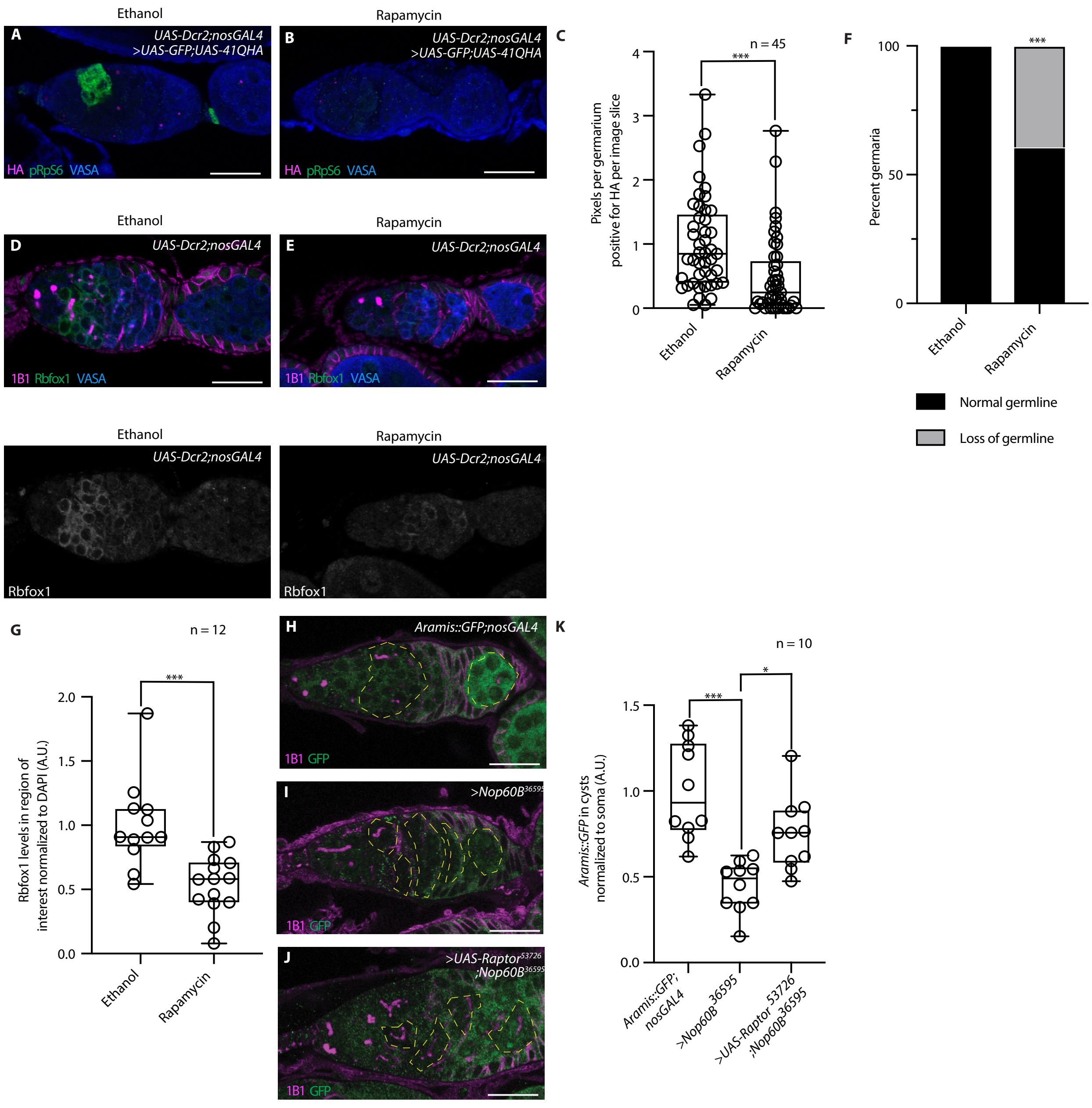

Fig S13. PolyQ proteins are sensitive to ribosome levels

(A, B) Poly41Q-HA reporter in ethanol (A) and rapamycin (B) treated ovaries stained with anti-HA (magenta), anti-pRpS6 (green) and anti-Vasa (blue). Scale bar is 20 µm.

(C) HA reporter quantitation using unpaired t-test (n = 45, *** p<0.0005).

(D, E) *UAS-Dcr2;nosGAL4* in ethanol (D) and rapamycin (E) treated ovaries stained with anti-1B1 (magenta), anti-Rbfox1 (green) and anti-Vasa (blue). Scale bar is 20 µm.

(F) Quantification of oogenesis defects in ethanol vs rapamycin treated ovariole. Statistical analysis performed with Fisher's exact test (ethanol treated n = 20, rapamycin treated n = 71, *** $p<0.0003$).

(G) Quantitation of Rbfox1 levels normalized to DAPI. Statistics performed were unpaired t-test (ethanol n = 12, rapamycin n = 12, ***, $p=0.0004$).

(H) *Aramis::GFP;nosGAL4, Nop60B* (I) and *UAS-Raptor;Nop60B* (J) ovaries stained with anti-1B1 (magenta) and anti-GFP (green). Scale bar is 20 µm.

(K) GFP quantitation from the cyst region using Dunnett's multiple comparisons post-hoc test after one-way ANOVA (n = 10 for all, *** $p<0.0001$, ns $p = 0.0118$).

Table S1: (A) PTM code for the modifications identified. The 1st column represents the Modomics code, the 2nd column represents the PTM name and the 3rd column the shortened modification name. (B) Summary of RNA PTM profiles obtained from GSCs, GSC daughters, cysts (early cysts), young wild type (later cysts and early egg chambers) and wild type (late-stage egg chambers). Each value represents the average and standard deviation of the respective relative abundances (AvP%, see Methods). A different shade of color was assigned only if the RNA PTMs relative abundance was statistically different from that of the GSCs input reference (1st column) with a p value not exceeding 0.05.

Table S2: (A) Excel spreadsheet of the RNA modification screen that contains the gene names, stock numbers, type of modification and phenotype. The raw number of germaria were counted. (B) The RNA modification screen represented as percent phenotypes.

Table S3: Summary of PTM profiles obtained. Each value represents the average and standard deviation of the respective relative abundances (AvP%, see Methods). A different shade of color was assigned only if the RNA PTMs relative abundance was statistically different from that of the cysts input reference (1st column) with a p value not exceeding 0.05.

Table S4: Spreadsheet of mRNA targets identified from pull-down utilizing pseudouridine antibody with a 2-fold cut off. Pseudourine pull-down followed by poly(A) selected mRNAs (IP) compared to poly(A) selected mRNAs from the input. (A) Genes that were lower than 2-fold enriched (B) genes that were higher than 2-fold enriched and (C) fold-enrichment values for all genes. It is possible only mRNAs that are highly pseudouridylated are pulled down and lowly pseudouridylated RNAs may not represented due to methodological lack of sensitivity.

Table S5: (A) MEME discriminative mode motif enrichment output of the 5' UTR, CDS and 3' UTR of genes that are lowly associated with ribosomes in germaria depleted of Nop60B. E-value, sites and width are provided for each identified motif. (B) MEME discriminative mode motif enrichment output of the 5' UTR, CDS and 3' UTR of genes highly associated with polysomes in germaria depleted of *Nop60B*. E-value, sites and width are provided for each identified motif.

Table S6: (A) Correlation plots comparing Ribo-Seq datasets showing high reproducibility between libraries. (B) Column A: mRNA targets identified by Ribo-Seq that contain the CAG motif. Column B: mRNAs containing a strict repeating CAG (no interruptions). Column C: locations of the CAG motif. Column D: length of the longest CAG repeat present in the mRNA or if there are other amino acid repeats present.

Table S7: (A) Find Individual Motif Occurrences (FIMO) output of QQQQQ motif search in genes that were lowly associated with polysomes in Nop60B depleted germaria. List of 181 unique genes that significantly contain a motif resembling QQQQQ. (B) All transcripts from the FIMO output of QQQQQ motif search in genes lowly associated with the polysome in Nop60B depleted germaria. Also provided are the p-value and matched motif sequences in each transcript. (C) Unique genes (3665) identified from FIMO output of 5-CAG motif search in genes with 1 or more transcripts per million in the cyst stage of development. (D) Output from FIMO of 5-CAG motif search in genes with 50 or more transcripts per million in the cyst stage of development. (E) Output from FIMO of 5-CAG motif search in genes with 1-50 transcripts per million in the cyst stage of development.

Primer List: (A) List of Stellaris *in situ* probes to detect *Rbfox1* mRNA. (B) List of Stellaris *in situ* probes used to detect *bru1* mRNA. (C) List of probes used for *Nop10*, *Nop60B* and *α-Tubulin at 84B* for qPCR analysis.
